# A small molecule targeting myoferlin exerts promising anti-tumor effects on breast cancer

**DOI:** 10.1038/s41467-018-06179-0

**Published:** 2018-09-13

**Authors:** Tao Zhang, Jingjie Li, Yuan He, Feifei Yang, Yun Hao, Wangrui Jin, Jing Wu, Zhenliang Sun, Yunqi Li, Yihua Chen, Zhengfang Yi, Mingyao Liu

**Affiliations:** 1 East China Normal University and Shanghai Fengxian District Central Hospital Joint Center for Translational Medicine, Shanghai Key Laboratory of Regulatory Biology, Institute of Biomedical Sciences and School of Life Sciences, East China Normal University, 200241 Shanghai, China; 2 Department of Orthopaedics, Shanghai General Hospital, Shanghai Jiao Tong University School of Medicine, 200080 Shanghai, China; 3 The Institute of Cell Metabolism and Disease, Shanghai General Hospital, Shanghai Jiao Tong University School of Medicine, 200080 Shanghai, China; 4 Shanghai University of Medicine & Health Sciences Affiliated Sixth Peopleʼs Hospital South Campus, Shanghai, 201499 China; 5 Center for Cancer and Stem Cell Biology, Institute of Biosciences and Technology, Texas A&M University Health Science Center, 77030 Houston, USA

## Abstract

Breast cancer is one of the most lethal cancers in women when it reaches the metastatic stage. Here, we screen a library of small molecules for inhibitors of breast cancer cell invasion, and use structure/activity relationship studies to develop a series of small molecules with improved activity. We find WJ460 as one of the lead compounds exerting anti-metastatic activity in the nanomolar range in breast cancer cells. Proteomic and biochemical studies identify myoferlin (MYOF) as the direct target of WJ460. In parallel, loss of MYOF or pharmacological inhibition of MYOF by WJ460 reduces breast cancer extravasation into the lung parenchyma in an experimental metastasis mouse model, which reveals an essential role of MYOF in breast cancer progression. Our findings suggest that MYOF can be explored as a molecular target in breast cancer metastasis and that targeting MYOF by WJ460 may be a promising therapeutic strategy in MYOF-driven cancers.

## Abstract (web summary)

Improved therapeutics are needed for treating breast cancer. Here they show the druggability of myoferlin with a small molecule inhibitor in breast cancer and demonstrate its anti-breast cancer effects in vitro and in vivo.

## Introduction

Breast cancer represents an aggressive disease with high prevalence that can develop invasive capability, then rapidly metastasize to other organs^1^. Metastatic disease is the final stage of breast cancer and the prognosis of metastatic breast cancer is extremely poor^2,3^. Therefore, developing effective therapeutics for preventing breast cancer metastasis is urgently needed.

In recent years, targeted therapies have led to spectacular progress in breast cancer therapy. Encouraging results have been observed with endocrine therapy and HER2-targeted therapy^4^. Regrettably, a significant fraction of patients still develop recurrence and distant metastases and eventually succumb to the disease. Basic research has contributed to a deeper understanding of the biology underpinning the malignant progression of breast cancer thus expanding the spectra of potential molecular targets. Currently, numerous studies have identified important oncogenic drivers that can be pharmaceutically targeted in the setting of metastatic breast cancer. Therapies developed to target phosphoinositide-3 kinase/AKT/mammalian target of rapamycin signaling significantly improved disease-free survival^5^. Other therapeutics such as cyclin-dependent kinase 4/6 inhibitors also showed promising antitumor activity in a phase III clinical trial examining patients with hormone receptor-positive metastatic breast cancer that had progressed on prior endocrine therapy^6^. Moreover, multiple lines of evidence support the existence of DNA repair deficiencies in lethal breast cancer. The success of poly ADP-ribose polymerase inhibitors in treating advanced breast cancer with DNA repair defects such as *BRCA1/2* mutations exemplify this^7^. In parallel, an ever-growing body of evidence supports the possibility that identifying the mechanisms underlying immune escape has potential to improve metastatic breast patient outcomes. MK-3475, an anti-PD1 antibody, showed therapeutic activity in patients with recurrent/metastatic triple-negative breast cancer (TNBC) in a phase I clinical study^8^. Nevertheless, these therapies are developed to perturb neoplastic growth, and despite the progress they made in metastatic breast cancer therapy, many patients will experience treatment failure. Therefore, additional therapies targeting the metastasis cascade should be considered.

Breast cancer metastasis is a complex process: local invasion by the primary tumor first occurs, invasive breast cancer cells then enter the circulatory system and overcome many obstacles to infiltrate distant organs, survive as disseminated seeds, and then grow at the distal site to form a metastasis^9^. The initial step in metastasis is that tumor cells achieve invasive capability^10^. Drugs that target invasion may reduce the incidence of metastatic disease.

In recent years, several groups of researchers have described the selective overexpression of myoferlin (MYOF) in breast carcinoma specimens^11,12^. MYOF may act as a key regulator in epidermal growth factor receptor (EGFR) degradation after its activation and internalization in breast cancer cells^12^. In addition, research has revealed that MYOF functions in breast cancer invasion and epithelial-to-mesenchymal transition (EMT), suggesting that MYOF may act as a modifier of breast cancer metastasis^13–15^. Another study unveiled a critical role of MYOF in TNBC metabolism and a positive correlation between MYOF expression level and TNBC metastasis^11^. Intriguingly, MYOF loss-of-function impairs breast cancer development in vivo^11^. These findings led to the hypothesis that targeting MYOF may impair breast cancer metastasis. Here types of small molecules with diaryl-thiazolidinone scaffold were identified in a screen of our in-house library against breast cancer metastasis, and WJ460, as one of the most potent leads, was confirmed using an in vitro invasion assay. WJ460 exhibited potent anti-metastatic activity against breast cancer in both spontaneous and experimental metastasis mouse models. We also identified MYOF as the direct target of WJ460. Collectively, our results demonstrated that WJ460 can serve as a first lead compound for the development of MYOF-targeted therapeutic agents and targeting MYOF by WJ460 may be a promising therapeutic strategy in MYOF-driven breast cancer.

## Results

### Discovery of WJ460

**Fig. 1:**
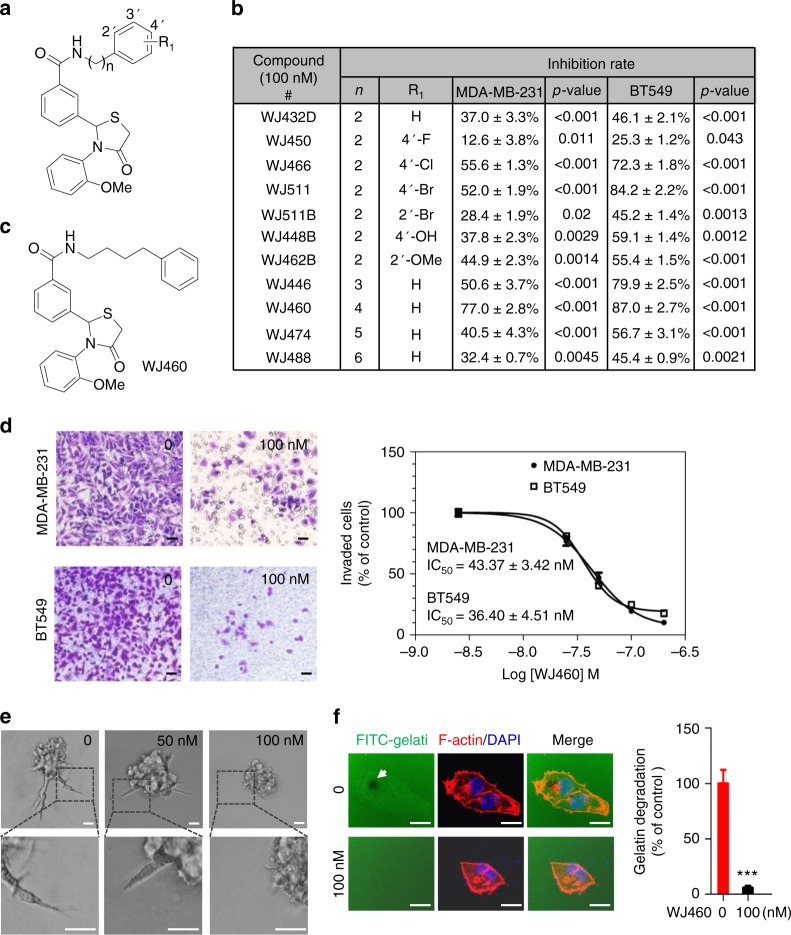
WJ460 inhibits breast cancer invasion in vitro. **a** Chemical structure of thiazolidinone derivative family. **b** Structure–activity relationship for thiazolidinone derivative analogs and inhibition rate on breast cancer cells (*n* = 3). **c** Chemical structure of WJ460. **d** MDA-MB-231 and BT549 cells were re-suspended in serum-free medium containing increasing concentrations of WJ460 and seeded into each transwell insert precoated with 10% matrigel. After 12 h, invading cells were stained with 0.1% crystal violet. Images were acquired using an inverted microscope (Olympus), with WJ460 dosage noted in upper right of each photomicrograph. WJ460 inhibited MDA-MB-231 and B549 cells invasion with an IC_50_ of 43.37 ± 3.42 nM and 36.40 ± 4.51 nM, respectively (*n* = 3). Scale bars, 50 μm. **e** 3D Matrigel invasion assay. MDA-MB-231 cells were plated above a layer of Matrigel and covered with growth medium containing 10% Matrigel and different concentrations of WJ460 (*n* = 3). Arrows, stellate invasive structure. Scale bars, 20 μm. **f** MDA-MB-231 cells were seeded on FITC-gelatin for 12 h. F-actin was stained with phalloidin (red) and nuclei with DAPI (blue). Areas of gelatin degradation appear as punctuate black areas beneath the cells. ***p < 0.001. Scale bars, 10 μm. Student’s *t* test, *n* = 3. In **b**–**f**, *n* indicates the number of independent experiments performed

To specifically identify inhibitors of breast cancer invasion, a canonical Matrigel-coated transwell invasion assay was first utilized. We screened our in-house small molecule library (>200 compounds with structural diversity) and found a series of 2-(3-(arylalkyl amino carbonyl) phenyl)-3-(2-methoxy-phenyl)-4-thiazolidinone derivatives that exhibited potent anti-invasion activity (Fig. 1a, b). To preliminarily investigate whether or not the substitution of terminal aromatic ring in WJ432D with electron-withdrawing groups and electron-donating groups would affect potency, we synthesized and evaluated the compounds WJ450–WJ462B listed in Fig. 1b. Notably, introduction of fluoro to the scaffold (WJ450) at the 4′-position caused a weak decrease in inhibitory activity compared to WJ432D. However, substituent with chloro or bromo (WJ466 or WJ511) showed better inhibitory activities. In addition, introduction of bromo (WJ511B) at the ortho-position caused a decrease in the inhibitory activities compared with WJ511; it seems that substituent at 4′-position might be more helpful to inhibitory activity than the one at 2′-position (WJ511 versus WJ511B). Furthermore, comparing compounds WJ448B and WJ462B to WJ432D, it could be found that the groups with electron donor property also show similar or a little increase to the inhibitory activity. Compounds WJ446–WJ488 were further synthesized in order to demonstrate the effect of the linker between the nitrogen atom and terminal phenyl ring. The results indicated that the length of methylene chain greatly affected the inhibitory activity; the lead compound WJ460 with four methylene chain was established as the most effective inhibitor among the investigated compounds (Fig. 1b, c; and the synthesis scheme is shown in Supplementary Fig. 1, 2). A more detailed cell-based Matrigel-invasion assay showed that WJ460 blocked breast cancer cell invasion compared with control condition (dimethyl sulfoxide (DMSO)) with IC_50_ values of 43.37 ± 3.42 nM in MDA-MB-231 and 36.40 ± 4.51 nM in BT549 cells (Fig. 1d). In addition, WJ460 treatment remarkably inhibited MDA-MB-231 and BT549 cells (Fig. 1d). Also WJ460 treatment remarkably inhibited MDA-MB-231 and BT549 cell invasion through Collagen I in a dose-dependent manner (Supplementary Fig. 3). Breast cancer cell invasiveness was also evaluated using a three-dimensional (3D) Matrigel invasion assay in which breast cancer cells were seeded on top of the Matrigel surface. MDA-MB-231 breast cancer cells invaded spontaneously into the Matrigel and showed multiple invasive stellate structures. However, few invasive structures were found in the WJ460 (100 nM) treated group (Fig. 1e). The ability of tumor cells to degrade extracellular matrix (ECM) is considered a key step promoting tumor invasion and metastasis. We next investigated whether WJ460 was associated with decreased breast cancer cells' ECM degradation. MDA-MB-231 cells were seeded on fluorescein isothiocyante (FITC)-conjugated gelatin matrix and exposed to different concentrations of WJ460. We found control cells markedly degraded ECM in 12 h, while cells in the WJ460-treated group failed to do so (Fig. 1f). Together, these results indicate that WJ460 inhibits breast cancer cell invasion with potent efficacy.

### The effect of WJ460 in a spontaneous metastasis model

**Fig. 2:**
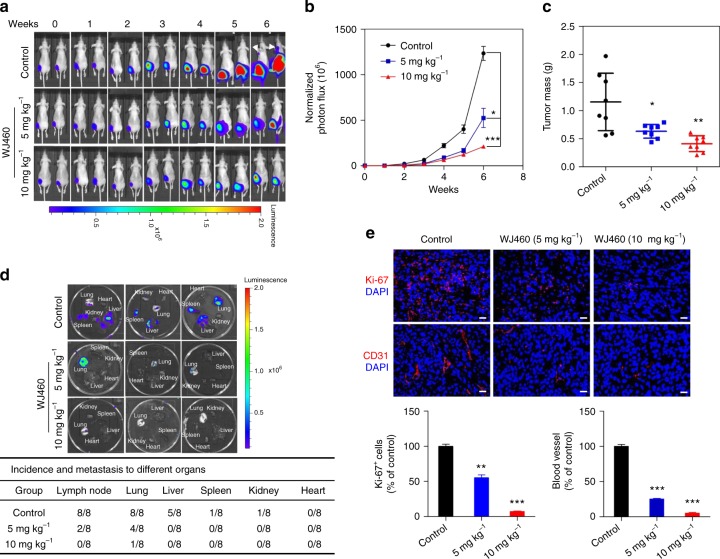
WJ460 exhibits potent antitumor activity in a spontaneous metastasis mouse model. **a** MDA-MB-231-Luciferase cells were implanted orthotopically in the mammary fat pad of female nude mice. When the tumor was palpable (day 7), mice were allocated into different groups according to the initial bioluminescence (*n* = 8). DMSO or WJ460 was administered intraperitoneally. Tumor growth was monitored weekly. Arrows, lymph node metastasis. **b** Quantification of bioluminescence. Data shown are mean ± s.d. *p < 0.05, ***p < 0.001. **c** When the experiment was terminated, all mice were sacrificed and the primary tumor was dissected and weighed. *p < 0.05, **p < 0.01. **d** Major organs were imaged for tumor presence shortly after harvesting. **e** Primary tumor sections were stained for Ki67 or CD31. Nuclei were counterstained with DAPI. Positive cells and blood vessel density were analyzed by the IPP software. Scale bars, 10 μm. Data shown are mean ± s.d. **p < 0.01, ***p < 0.001

We next examined the in vivo efficacy of WJ460 using a spontaneous breast cancer metastasis model. Upon orthotopic injection, the Luciferase-labeled MDA-MB-231 (MDA-MB-231-Luciferase) cells grow aggressively in the mouse mammary fat pad and spontaneously metastasize to distant organs. MDA-MB-231-Luciferase cells were inoculated into the #4 mammary gland of female nude mice and monitored overtime by bioluminescence. Of note, WJ460 resulted in substantially decreased tumor growth versus vehicle treatment (Fig. 2a–c). When the experiment was terminated, major organs in each group were dissected and examined for metastases. MDA-MB-231 cells in the control group effectively colonized lymph nodes, lungs, livers, spleens, and kidneys, whereas cells in the WJ460-treated groups displayed limited metastatic propensity (Fig. 2d). Examination of harvested tumors also indicated reduced tumor proliferation caused by WJ460, as evaluated by anti-Ki67 staining (Fig. 2e, upper panel). Using blot arrays that detect the phosphorylation of 42 different receptor tyrosine kinases (RTKs), we found that WJ460 blocked serum-induced activation of several kinases, such as fibroblast growth factor receptor 1, vascular endothelial growth factor receptor 2 (VEGFR2), and Tie2, suggesting that WJ460 may block metastasis through inhibiting RTK signaling in breast cancer cells (Supplementary Fig. 4a). Breast tumor growth and distant metastasis are associated with angiogenesis and VEGF plays a critical role in this process^16^. To detect whether WJ460 triggers anti-angiogenic activity, we examined VEGF-induced capillary tubule formation. We found that WJ460 significantly suppressed capillary-like tube formation of human umbilical vein endothelial cells (HUVECs) (Supplementary Fig. 4b). A western blot assay showed that addition of WJ460 diminished the level of phospho-VEGFR-2 and total VEGFR-2 (Supplementary Fig. 4c). We also evaluated tumors harvested from mice by immunostaining using the angiogenesis marker CD31. A marked decrease in intraneoplastic vascular density was observed in tumors from WJ460-treated mice versus control mice, indicating that administration of WJ460 resulted in reduced tumor vasculature (Fig. 2e, lower panel). Furthermore, administration of WJ460 induced no apparent toxicity and no changes in body mass and major organ behavior were observed (Supplementary Fig. 5a, b). Together, we conclude that WJ460 inhibits breast tumor growth, angiogenesis, and spontaneous metastasis.

### WJ460 suppresses breast cancer pulmonary metastasis in vivo

**Fig. 3:**
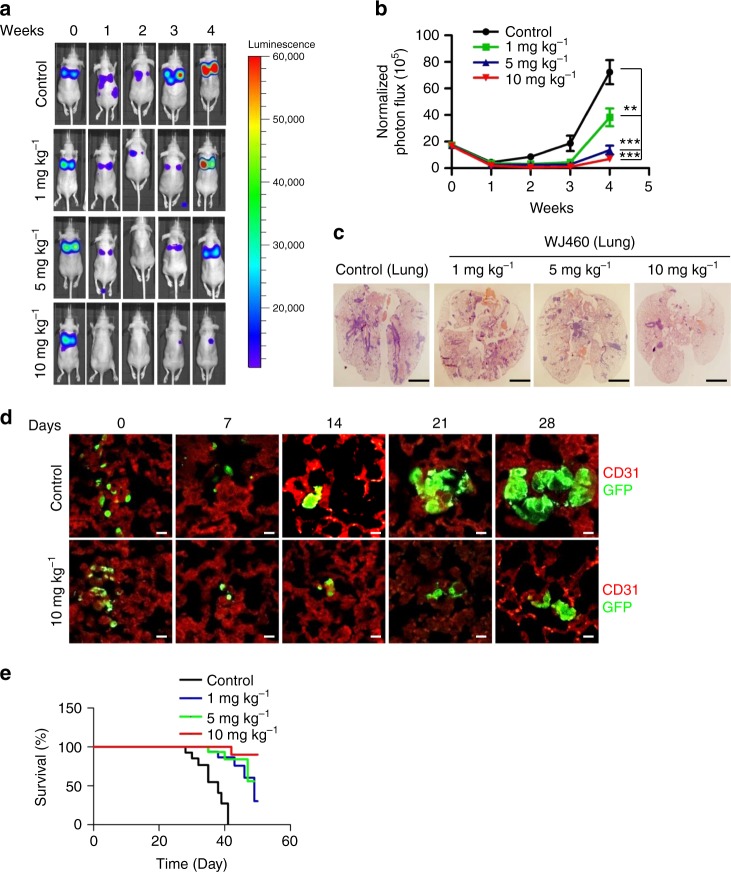
WJ460 abrogates breast cancer lung extravasation and colonization. **a** MDA-MB-231-Luciferase cells were inoculated intravenously into female nude mice. DMSO or WJ460 was injected intraperitoneally on day 0. Lung metastasis was monitored by bioluminescence using an in vivo imaging system. **b** Quantification of bioluminescence. *n* = 6 mice per group. **c** H&E staining of lungs from the indicated groups upon harvest (day 28). Scale bars, 0.4 cm. **d** Mice were intravenously injected with MDA-MB-231-Luciferase-GFP cells and sacrificed at the indicated times. Lungs were stained for CD31 (red) and GFP (green). Confocal images were acquired. Scale bars, 10 μm. *n* = 6 mice per group. **e** Overall survival rate of mice in different treatment groups. *n* = 8 mice per group. **p < 0.01, ***p < 0.001

Since we observed inhibition of primary breast tumor growth upon WJ460 treatment, we next sought to identify whether it would directly inhibit metastatic growth apart from effects on the primary tumor. We tested this using a mouse experimental metastasis model. MDA-MB-231-Luciferase cells were introduced intravenously into athymic nude mice (*n* = 6) and lung metastasis was monitored weekly by an in vivo image system and histological examination. Intraperitoneal injection of WJ460 caused significant inhibition of breast cancer pulmonary metastasis in a concentration-dependent manner (Fig. 3a–c). To analyze lung metastasis events during the experimental period after tumor cell inoculation, whole lungs were freshly removed and scanned by confocal microscopy. Confocal images showed that similar numbers of cells were present in the lungs shortly after injection (day 0). Only a fraction of breast cancer cells in WJ460-treated mice managed to invade into the stroma of the lungs (day 7). These result indicated that cancer cells that failed to metastasize hardly survived and WJ460 may have impaired the ability of MDA-MB-231 cells to survive in the lungs. From around day 14, a small portion of MDA-MB-231 cells in the control group started to proliferate, whereas outgrowth was impaired by WJ460 (bioluminescence signals in the 5 mg kg^−1^ as well as 10 mg kg^−1^ treated group can hardly be detected). By day 28, breast cancer cells reinitiate their proliferative programs at metastatic sites (lung) to generate macroscopic neoplastic growths. Metastatic foci in control mice became large and had undergone neoangiogenesis but those in the WJ460-treated group remained relatively small (Fig. 3d). In addition, mouse overall survival in the WJ460-treated groups was greatly increased (Fig. 3e). We conclude that WJ460 inhibited metastatic extravasation and outgrowth of breast cancer.

### Identification the potential target of WJ460

**Fig. 4:**
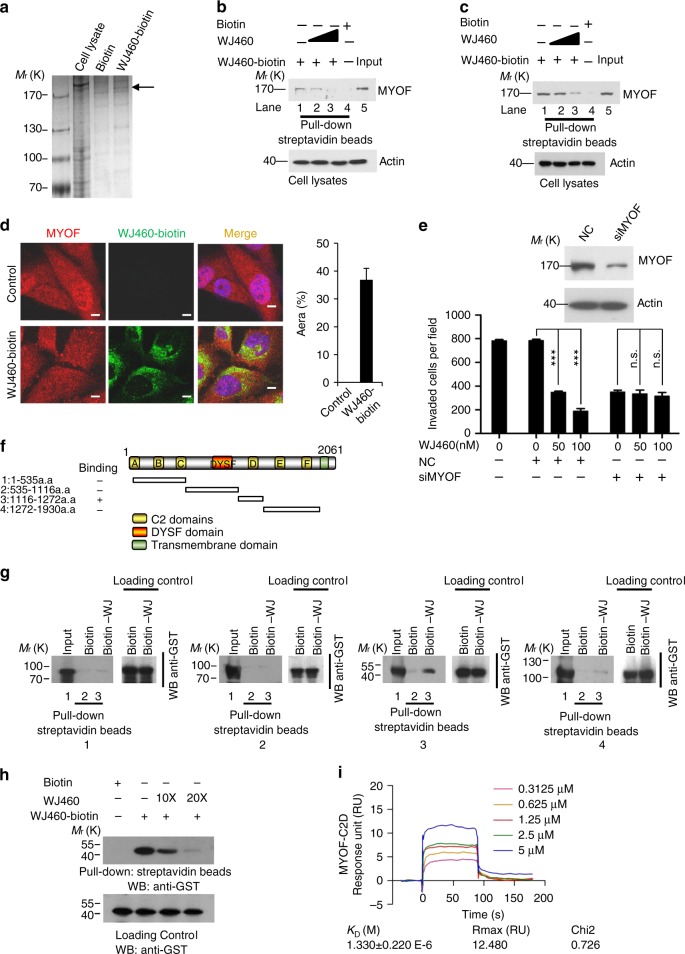
WJ460 directly targets MYOF. **a** MDA-MB-231 cell lysates were incubated with free biotin or WJ460–biotin. Protein affinity pull-down assay was performed and the precipitated proteins were separated by SDS-PAGE. The gel was processed by silver staining (*n* = 4). **b** Exogenous competition assay. MDA-MB-231 cell lysates were incubated with biotinylated-WJ460 (10 μM) or biotin in the presence or absence of 10 (100 μM) or 20 (200 μM) fold WJ460. The biotinylated-WJ460-bound protein was precipitated by streptavidin beads and immunoblotted with an antibody against MYOF (*n* = 3). **c** Exogenous competition assay. CHO cells were forced to express human MYOF. Cells overexpressing MYOF were lysed and lysates were incubated with biotinylated-WJ460 (10 μM) or biotin (10 μM) in the absence or presence of 10 (100 μM) or 20 (200 μM) fold WJ460. The mixtures were blotted for MYOF (*n* = 3). **d** MDA-MB-231 cells were treated with or without biotinylated-WJ460. Cells were then stained anti-MYOF (labeling MYOF, red) and streptavidin-FITC (labeling WJ460–biotin, green). Scale bars, 10 μm. *n* = 3. **e** Transwell invasion assay. Cells were transfected with scrambled siRNA or MYOF siRNA, and then treated with WJ460 (*n* = 3). Data shown are mean ± s.d. ***p < 0.001, n.s., not significant. **f** Schematic of constructs with different functional domains of MYOF. **g** GST-tagged deletion constructs were expressed in BL21 cells. The expressed proteins were incubated with WJ460–biotin or biotin and blotted by anti-GST (*n* = 3). **h** Construct containing MYOF C2D was expressed in BL21 cells and the competition assay was performed (*n* = 3). **i** Surface plasmon resonance analysis of interactions between WJ460 and MYOF (*n* = 3). In each panel, *n* indicates the number of independent experiments performed

Our data demonstrated that WJ460 is a potent inhibitor of breast cancer tumor growth and metastasis in vitro and in vivo. We next sought to explore the potential targets for WJ460 that contribute to its metastasis inhibitory capability. Based on the results of structure–activity relationship of this series of compounds, we engineered a biologically active WJ460–biotin conjugate (Supplementary Fig. 6a, b) and used this compound as an affinity matrix to identify proteins that bind to WJ460. Lysates of MDA-MB-231 cells were incubated with WJ460–biotin before being precipitated using streptavidin beads. Precipitated biotin–streptavidin complexes were processed on sodium dodecyl sulfate-polyacrylamide gel electrophoresis (SDS-PAGE) and silver stained. A protein of about 230 kDa (as indicated by the arrow head) was precipitated by WJ460–biotin but not by free biotin (Fig. 4a). Mass spectrometric analysis indicated that this 230 kDa protein was human MYOF (Supplementary Table 1). MYOF plays a pivotal role in breast cancer invasion and metastasis^14^. To confirm that MYOF is a WJ460-binding protein, an endogenous affinity pull-down assay was performed with WJ460–biotin and WJ460 using an antibody against MYOF. As shown in Fig. 4b, endogenous MYOF in MDA-MB-231 cells could be precipitated by WJ460–biotin. Concomitantly, increasing amounts of WJ460 competitively inhibited the binding of MYOF to WJ460–biotin. Human MYOF was also constitutively overexpressed in Chinese hamster ovary (CHO) cells (Supplementary Fig. 6c). An exogenous affinity pull-down assay was then carried out and similar results were obtained as the exogenous assay (Fig. 4c). In accordance with this result, confocal microscopic analysis revealed that MYOF and WJ460–biotin colocalize in the cytoplasm (Fig. 4d). A transwell invasion assay showed that treatment with MYOF small interfering RNA (siRNA) but not scrambled control siRNA decreased the invasion of MDA-MB-231 cells; importantly, MYOF knockdown cells were not sensitive to WJ460 treatment compared with control cells (Fig. 4e), indicating that MYOF was a key target of WJ460 for inhibition of invasion. This result was confirmed in our animal experiment (Supplementary Fig. 7). In summary, our data demonstrated that MYOF is a direct protein target of WJ460.

### MYOF C2 domain (C2D) is required for its interaction with WJ460

C2Ds are regulatory sequence motifs and consist of about 120 amino acids that are implicated in Ca^2+^ binding and protein–protein interaction^17–19^. Given that MYOF is a multi-C2D-containing protein, we investigated the binding properties of C2Ds and WJ460. At least six C2Ds were predicted in MYOF (Fig. 4f) and MYOF deletion constructs containing different C2Ds were expressed as glutathione *S*-transferase (GST) fusion proteins in *Escherichia coli*. WJ460 demonstrated direct binding to the C2D but its ability to interact with other regions of MYOF was nearly undetectable (Fig. 4f, g). To confirm that WJ460 binds to the C2D of MYOF, binding competition assay was performed using a GST fusion protein. As shown in Fig. 4h, increasing concentrations of WJ460 reduced the amount of C2D precipitated by WJ460–biotin. Finally, the binding between WJ460 and MYOF C2D was examined by surface plasmon resonance (SPR) assay. Binding of MYOF C2D (molecular weight ~44 kDa) with WJ460 showed time-dependent saturation and the *K*_D_ value was about 1.330 ± 0.220 μM (Fig. 4i). To improve the robustness of this experiment, we used a negative molecule (YQ268A) instead of WJ460 to performed SPR experiment. We found that YQ268A could not bind to MYOF C2D (Supplementary Fig. 8a). Collectively, our data indicated that MYOF directly interacts with WJ460 by its C2D. In order to elaborate which amino acid residue/residues is/are mainly responsible for the binding between WJ460 and MYOF C2D, a homology modeling was used in constructing a 3D model of C2D of MYOF protein since the structure of MYOF C2D has not yet been resolved up to date. In this preliminary docking model, the docking pattern showed that WJ460 can interact with multiple amino acid residues in the C2D, including Cys5 (that is, Cys1143 in full-length MYOF), Val73 (Val1211 in full-length MYOF), Met75 (Met1213 in full-length MYOF), Ser92 (Ser1230 in full-length MYOF), Phe94 (Phe1232 in full-length MYOF), Glu103 (Glu1241 in full-length MYOF), Leu111 (Leu1249 in full-length MYOF), His113 (His1251 in full-length MYOF), and Val127 (Val1265 in full-length MYOF) (Supplementary Fig. 9a, b). Among these functional amino acids, we found that His113 (His1251 in full-length MYOF) has a π–π interaction with the phenyl ring, which plays a crucial role in the binding of WJ460. In addition, Glu103 (Glu1241 in full-length MYOF) may interact with the amide of phenyl ring of WJ460 via hydrogen bond interaction, and Phe94 (Phe1232 in full-length MYOF) and Ser92 (Ser1230 in full-length MYOF) may also have molecule–molecule interaction with WJ460. Therefore, we chose the His113, Glu103, Phe94, and Ser92 sites in the C2D for mutation to verify which regions WJ469 binds to. As shown in Supplementary Fig. 9b, mutation of C2D His113 (His1251 in full-length MYOF) to Ala lost its ability to bind to biotin–WJ460. Additionally, C2D His113 Ala mutant rendered MYOF resistant to WJ460 treatment (Supplementary Fig. 9c). Collectively, our data indicated that MYOF directly interacts with WJ460 by its C2D.

### WJ460–MYOF interaction hampers the proper function of MYOF

**Fig. 5:**
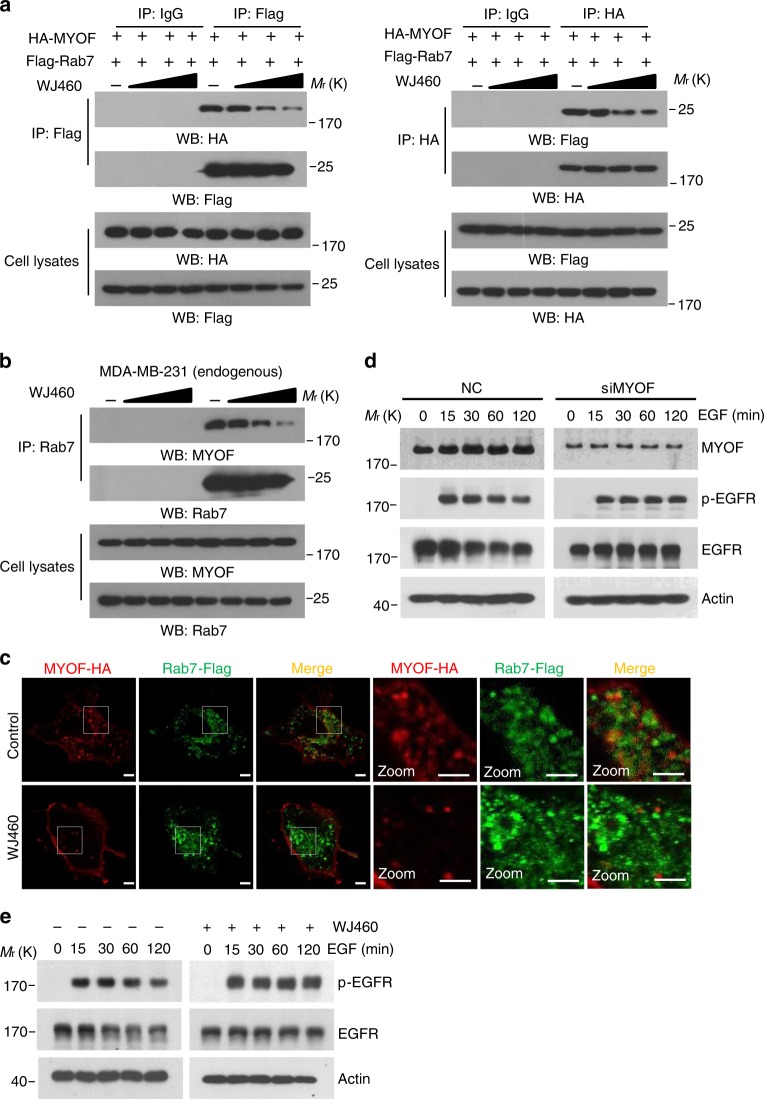
WJ460 facilitates the dissociation of MYOF in Rab7-positive late endosomes. **a** HA-MYOF and Flag-Rab7 were co-transfected in 293T cells. Cells were then exposed to increasing concentrations of WJ460 (50 nM, 100 nM and 200 nM). Immunoprecipitation assays were performed using anti-Flag M2 gel or anti-HA gel (*n* *=* 3). **b** MDA-MB-231 cells were treated with increasing concentration of WJ460 (50 nM, 100 nM and 200 nM) and a co-IP assay was performed with anti-Rab7 antibody (*n* = 3). **c** 293T cells co-transfected with HA-MYOF and Flag-Rab7 were treated WJ460 (100 nM). Cells were then fixed and stained for HA (red) and Flag (green) (*n* = 3). Scale bars, 10 μm. **d** Time-dependent phospho-EGFR and EGFR expression following EGF stimulation with or without myoferlin knockdown. **e** Time-dependent phospho-EGFR and EGFR expression after EGF stimulation with or without WJ460 exposure (*n* = 3). In each panel, *n* indicates the number of independent experiments performed

MYOF belongs to the ferlin family, which is an evolutionarily conserved family of vesicle fusion proteins that contributes to endocytosis^20,21^. Alterations in the endocytic pathway (that is, internalization and recycling routes) are frequently observed in cancer cells; aberrant endocytosis of transmembrane proteins leads to malignant transformation, thus promoting tumor cell proliferation, migration, and invasion^11,22^. Amplification of MYOF has been implicated in breast cancer. MYOF is a regulator of breast cancer invasion as well as RTK recycling^15^. Further study indicated that MYOF acts as a key regulator in EGFR degradation after its activation and internalization in breast cancer cells^12^. These studies raised the possibility that MYOF may serve as a breast cancer-related protein via controlling tumor-associated endocytic processes. Recently, MYOF was found to localize mainly within Rab7-positive late endosomes in the endocytic system where it functions in cargo recycling^23^. We then hypothesized that the interaction between WJ460 and MYOF is responsible for the dissociation of MYOF from Rab7-positive late endosomes, therefore blocking its proper function. Hemagglutinin (HA)-MYOF and Flag-Rab7 were transiently co-transfected in 293T cells. Immunoprecipitation with anti-Flag M2 affinity gel showed that MYOF specifically interacted with Rab7; however, MYOF/Rab7 complex formation was dose-dependently impaired upon exposure to WJ460 (Fig. 5a, left panel). This result was further verified through reciprocal immunoprecipitation analysis by using an anti-HA affinity gel (Fig. 5a, right panel). Similar results were observed in an endogeneous co-immunoprecipitation assay with MDA-MB-231 cells (Fig. 5b). We next used an immunofluorescence staining assay to detect whether WJ460 reduced MYOF in late endosomes. As shown in Fig. 5c, MYOF displayed a strong co-localization with Rab7 in control cells, and the co-localization was blocked in the presence of WJ460. However, the protein level of MYOF remained relatively constant after WJ460 exposure. In parallel, we examined RTK recycling by investigating the phosphorylated forms of several RTKs. RTK recycling was interrupted (Supplementary Fig. 4) and EGF-induced EGFR degradation was impaired after knockdown of MYOF or WJ460 treatment (Fig. 5d, e and supplementary Fig. 10), which is in accordance with previous studies. MYOF was shown to affect the functional assembly of caveolin into caveolae without influencing clathrin-mediated process^12^. We observed that caveolin foci in WJ460-treated cells were larger in size (Supplementary Fig. 11a). Also, MYOF was found to regulate VEGFR2 activity through preventing its polyubiquitination and proteasomal degradation. We found that proteasome inhibitor MG132 treatment can partly rescue WJ460-induced VEGFR2 degradation and WJ460 dramatically increased the ubiquitination level of VEGFR2 (Supplementary Fig. 11b, c). These data confirmed our hypothesis that WJ460 facilitated the dissociation of MYOF from Rab7-positive late endosomes and blocked its proper function in the endocytic pathway.

### A role of MYOF in the pathogenesis of breast cancer

Previous work indicated that MYOF is overexpressed in breast cancer^12^. To further explore the potential role of MYOF in breast cancer tumorigenesis, we analyzed the baseline MYOF expression in 90 human breast cancer biopsy samples and 10 adjacent non-transformed specimens. MYOF expression showed a positive correlation with breast cancer progression (Supplementary Fig. 12a). Our results indicated that MYOF expression in breast cancer samples was much higher than in normal tissues. The protein level of MYOF tends to increase in metastatic breast cancers. Notably, tumors of patients with metastatic breast cancer have higher MYOF levels (52.5%) than adjacent non-cancerous tissues (12.5%) and tumors from patients with in situ breast carcinoma (25%) (Supplementary Fig. 12b). Together, these data suggested that a high MYOF level is associated with increased aggressiveness of breast cancer.

### WJ460 reverses EMT in breast cancer cells

**Fig. 6:**
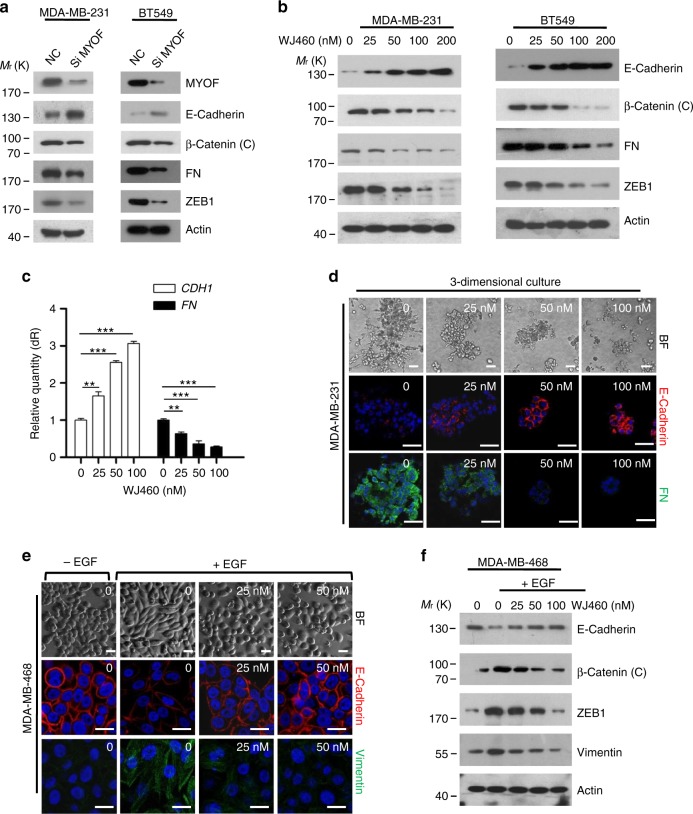
WJ460 reverses EMT in breast cancer cells. **a** MYOF ablation in the indicated breast cancer cells increased the expression of epithelial marker E-cadherin (Cytosolic extracts), whereas mesenchymal markers cytosolic β-catenin, fibronectin and ZEB1 were downregulated (*n* = 3). **b** Western blot analysis of EMT markers after WJ460 treatment (*n* = 3). **c** Quantitative RT-PCR analysis of EMT markers after WJ460 treatment (*n* = 3). Data shown are mean ± s.d. **p < 0.01, ***p < 0.001. **d** MDA-MB-231 cells were seeded on a layer of Matrigel. Growth medium containing 10% Matrigel and different concentrations of WJ460 was added on top of the cells. After 4 days, cells were extracted by PBS-EDTA and replated onto coverlips. Cells were then fixed and stained for E-cadherin (red) and fibronectin (green) (*n* = 3). Scale bars, 50 μm. **e** MDA-MB-468 cells were treated with WJ460 and EGF or not. Four days after treatment, cells were stained for E-cadherin (red) and Vimentin (green) (*n* = 3). Scale bars, 20 μm. **f** Western blot analysis of MDA-MB-468 cells undergoing EGF-induced EMT (*n* = 3). In each panel, *n* indicates the number of independent experiments performed

Derailed endocytosis results in several hallmarks of cancer including enhanced invasiveness and EMT^24^. EMT endows cancer cells with an invasive phenotype and EMT is also essential in other stages of tumor metastasis^25^. WJ460 binds to MYOF to repress its role in the endocytic pathway. We wondered whether MYOF was involved in EMT. Genetic ablation of MYOF promotes a mesenchymal–epithelial transition in breast cancer cells. In accordance with these data, we found that MYOF deletion increased the abundance of E-cadherin protein but decreased cytosolic β-catenin, fibronectin (FN), and ZEB1 (Fig. 6a). Concomitantly, knockdown of MYOF using specific short hairpin RNA (shRNA) hairpins also showed reduced metastasis in the lungs (Supplementary Fig. 7a, b). To determine whether exposure to WJ460 resulted in the same effects, breast cancer cells were treated with different concentrations of WJ460. Our data indicated that WJ460 could reverse the highly metastatic mesenchymal-like MDA-MB-231 and BT549 cells back to an epithelial state. Expression of the epithelial marker E-cadherin was significantly upregulated in WJ460-treated cells. Conversely, expression of mesenchymal markers cytosolic β-catenin, FN, and ZEB1 were drastically reduced in the presence of WJ460 (Fig. 6b, c). Next, we used an in vitro 3D culture assay to further evaluate the role of WJ460 in breast cancer EMT for the following reasons: (1) an in vitro 3D culture assay is suitable and useful to discriminate cellular and behavioral aspects of EMT and (2) 3D cultures can be extracted to assess EMT-related markers^26,27^. We found that control cells generated branched organoid morphologies when grown in 3D culture, whereas WJ460-treated cells adopted an epithelial morphology concomitant with increased E-cadherin and loss of FN expression (Fig. 6d). Soluble growth factors such as transforming growth factor-β, platelet-derived growth factor, and EGF have been described to trigger EMT^28^. In an EGF-induced EMT model, MYOF ablation impairs the ability of breast cancer cells to undergo EMT. In our study, EGF shifted the epithelial-like MDA-MB-468 breast cancer cells to a mesenchymal phenotype and WJ460 compromised this ability (Fig. 6e). Additionally, WJ460-treated cells exhibited reduced the levels of mesenchymal markers and gained the expression of epithelial markers (Fig. 6e, f). In sum, these studies indicated that WJ460 could reverse breast cancer cells back to an epithelial state.

### WJ460 attenuates the metastatic ability in other tumors

**Fig. 7:**
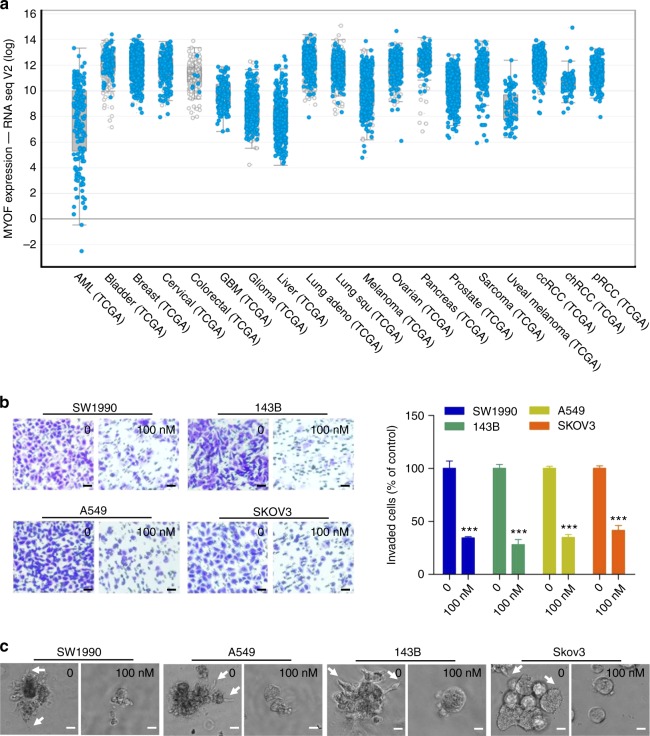
WJ460 attenuates the metastatic ability of cell lines derived from other tumors. **a** The expression of MYOF gene was detected in various cancers using cBioportal online Web resource (cBioportal for Cancer Genomics). **b** Transwell invasion assay. Various cancer cell lines were re-suspended in serum-free medium containing increasing concentrations of WJ460 and seeded into transwell inserts precoated with 10% matrigel. After 12 h, invaded cells were stained with 0.1% crystal violet. Images were acquired using an inverted microscope (Olympus) (*n* = 3). Scale bars, 50 μm. Data shown are mean ± s.d. ***p < 0.001. **c** 3D on-top culture assay. Cells were plated above a layer of Matrigel and covered with growth medium containing 10% Matrigel and the indicated concentration of WJ460 (*n* = 3). Arrows, stellate invasive structure. Scale bars, 20 μm. In **b**, **c**, *n* indicates the number of independent experiments performed

We next investigated whether WJ460 exhibits anti-metastatic potential in other tumor types with high MYOF expression. It was intriguing to find that MYOF is also highly expressed in several other tumor types such as lung adenocarcinoma, pancreatic adenocarcinoma and sarcoma when we analyzed the The Cancer Genome Atlas and cBioPortal database (Fig. 7a). We then selected four non-breast cancer cell lines (SW1990 pancreatic cancer cells, 143B sarcoma cells, A549 lung cancer cells, and SKOV3 ovarian cancer cells) representing other human tumor types with high MYOF levels. WJ460 treatment led to decreased invasion through Matrigel in all four cell lines derived from other tumor types with high MYOF (Fig. 7b). Finally, we seeded four non-breast cancer cell lines in 3D Matrigel culture and treated with or without WJ460. All four cell lines formed invasive stellate structures into the Matrigel, indicating their metastatic propensity; however, WJ460 exposure caused less invasive behavior with less invasive stellate structure formation in all the tested cell lines (Fig. 7c). Collectively, these observations suggest that WJ460 attenuates the metastatic ability of other tumor types that express a high level of MYOF.

## Discussion

Current breast cancer therapeutic intervention strategies aim to eradicate cancer cells. The efficacious of most therapeutic agents heavily depend on their genotoxic/cytotoxic capability. However, cancer cells often acquired the ability to circumvent the death programs caused by therapeutic agents and thereby led to relapse or clinically detectable metastasis^29^. Breast cancer metastasis is responsible for the vast majority of cancer-related mortality and still remains a major hurdle in breast cancer therapy. This warrants the development of more specific approaches targeting tumor metastasis. Our findings introduce a small molecule, WJ460, with potent anti-breast cancer metastasis effect in vitro and in vivo.

During the past decades, the metastatic cascade has been well illuminated. Molecular technologies such as DNA microarrays and genome sequencing have facilitated the identification of potential proteins that modulate metastasis^30^. Using a combination of mathematical modeling and in vitro invasion assays, researchers uncovered that MYOF might act as a critical mediator in breast cancer metastasis. In one subsequent proteomic profiling analysis, MYOF was identified as a pivotal breast cancer-related protein^12^. In the present study, we found that WJ460 interacts directly with MYOF using synthesized biotin-labeled WJ460 as a probe. Currently, MYOF is reported to localize in Rab7-positive late endosomes, suggesting a potential role in the endocytic pathway^23,31^. It is now well established that the endocytic compartment and endocytic pathway contribute to processes driving malignant transformation and cancer cell motility. Our further investigation showed that WJ460 binding to MYOF sequestered MYOF from Rab7-positive late endosomes, thereby impairing the proper function of late endosomes and the endosomal system in cancer cells. This is in accord with reports that genetic knockdown of MYOF in TNBC cells leads to dysfunction of the late endosomes and fatty acid metabolism^11^. As a consequence, breast cancer metastatic capacity is largely impeded.

Defective endocytosis frequently leads to enhanced invasiveness and EMT. Our work demonstrated that WJ460 blocked breast cancer invasion and reversed highly metastatic mesenchymal-like breast cancer cells back to an epithelial state, suggesting that WJ460/MYOF complex formation may impede the role of MYOF in invasion. Furthermore, our finding of reduced RTK internalization following WJ460 treatment supports the hypothesis that WJ460 blocks endocytosis in a MYOF-dependent manner. However, deep studies are limited by several conundrums: (1) the precise molecular role of MYOF in the endocytic system still remains unveiled; (2) whether MYOF is involved in the sorting process of ECM components or adhesion molecules and thus facilitates cancer cell metastasis need further study. Our work demonstrated that WJ460 regulates metastatic-related biological processes closely linked to MYOF. Previous studies reported that MYOF is involved in RTKs' recycling (VEGFR2, e.g.) and MYOF depletion reduced VEGF exocytosis by cancer cells^15,32^. Interestingly, our in vitro and in vivo experiments showed that WJ460 suppressed capillary-like tube formation and neoangiogenesis. Hence, to some extent, the present work provides an opening for a mechanistic dissection of how WJ460 inhibits breast cancer metastasis.

Several additional biological implications emerge from our present work. MYOF was first identified as a regulator for muscular dystrophy and subsequent studies implicated it in vesicle-related processes, including endocytosis and vesicle transport^20,33^. More recently, mounting evidence supports an important role of extracellular vesicles (EVs) in tumor growth and metastasis. Cancer-derived EVs can interact with target cells by their surface membrane proteins and they can also be internalized into target cells via endocytosis. Release of EVs may transfer signaling molecules into recipient cells to exert phenotypic or functional regulation^34^. It has been shown that cancer-derived EVs transfer angiogenic proteins to endothelial cells and thus induce angiogenesis^34,35^. In parallel, EVs secreted from cancer cells are involved in the formation of the pre-metastatic niche at distant organs^36^. Recently, Turtoi and his colleagues recently have revealed an important function of MYOF in exosome biology in breast and pancreatic cancers; they demonstrated that MYOF is an exosomal protein and genetically silencing MYOF reduces both the size and loaded cargo in tumor-derived exosmose^31^. Given its exosome-related function, MYOF may act as a candidate protein for EV-modulated tumor growth and spread in breast cancer and small molecule compounds targeting MYOF (e.g., WJ460) seem likely to play an important role in EV-dependent tumor progression.

In recent years, the critical role of breast cancer stem cells (BCSCs) in metastasis has been increasingly recognized^37^. There is also circumstantial evidence that EMT endows cells with stem-like traits. We show that WJ460 could reverse mesenchymal-like MDA-MB-231 cells back to an epithelial state and also WJ460 blocked growth factor-induced EMT. This exposes the possibility of evaluating the effect of MYOF and WJ460 on BCSCs. Concomitantly, BCSCs are deemed to confer resistance to chemotherapy drugs, which is another challenge for breast cancer therapy^38^. Therefore, another question that arises is whether WJ460 could overcome chemotherapy resistance.

Tumor metastasis is a multistep process involving metastasis formation and colonization. Our work shows that WJ460 impedes breast cancer cell invasion and extravasation into the lung parenchyma, supporting the inhibitory capability of WJ460 in metastasis formation. Approximately one third of breast cancer patients harbor metastases in lymph nodes at the time of surgery, which highlights the necessity of drugs targeting metastatic colonization. Our current findings demonstrated that WJ460 modifies the ability of breast tumor cells to proliferate in metastatic lesions (Fig. 3). Although further studies are needed to reinforce these data, our results, to a certain extent, suggest that WJ460 may inhibit metastatic seeding and outgrowth.

In conclusion, our studies identified a metastasis-preventive compound, WJ460, and provide mechanistic insights into the link between WJ460 and metastasis. Our findings demonstrate that WJ460 blocks breast cancer metastasis through directly targeting MYOF. Results in the present work may reflect the role of MYOF in tumor metastasis and this raises the possibility to assess the clinical association of MYOF and metastasis. Our results also provide opportunities for developing MYOF-targeted agents using WJ460 as a lead compound.

## Methods

### Cell culture

MDA-MB-231, BT549, MDA-MB-468 breast cancer cells, 293T, 143B (human osteosarcoma cells), SKOV3 (human ovarian cancer cells), DU145 (human prostate cancer cells), and SW1990 (human pancreatic cancer cells) were purchased from ATCC (Manasseh's, VA, USA). Cells were maintained in L-15 or in Dulbecco’s modified Eagle’s medium (DMEM) supplemented with 10% fetal bovine serum (FBS) and 1% penicillin/streptomycin. MDA-MB-231-LUC was cultured in Minimum Essential Medium supplemented with 10% FBS and non-essential amino acids^39^. HUVECs (ScienCell Research Laboratories, San Diego, CA, USA) were purchased from Science Research Laboratories and cultured in complete ECM (ScienCell) supplemented with 5% FBS. CHO cells were maintained in DMEM supplemented with 10% FBS, 2 mM L-glutamine, 1 mM sodium pyruvate, and 100 μM non-essential amino acids. All cells were maintained at 37 °C under a humidified 5% CO_2_ incubator. All cell lines were periodically monitored for mycoplasma contamination.

### Chemicals

WJ460 was synthesized as described in Supplementary Information. Compound stock solutions were prepared in DMSO at a concentration of 100 mM and stored at −20 °C.

### Transwell invasion assay

Transwell invasion assay was performed following the manufacturer’s instructions with modifications. Breast cancer cells were resuspended in medium with test compounds and seeded on transwell filters (8 μm pore size; Millipore) precoated with Matrigel or Collagen I. After 12 h, cells on the top side of the filters were wiped by cotton swaps. Cells on the lower side were then fixed in 4% paraformaldehyde and stained with 0.1% crystal violet. Images were taken under an inverted microscope (Olympus).

### 3D on-top culture

3D culture assay was conducted as described^40^. Briefly, MDA-MB-231 breast cancer cells were seeded on a 48-well plate coated with a thin layer of Matrigel. Thirty minutes post-seeding, medium containing 10% Matrigel and different concentrations of WJ460 were added to the plated culture. The culture was maintained for 4 days and the on-top Matrigel–medium mixture with or without WJ460 was replaced every 2 days.

### In situ gelatin degradation assay

In brief, coverslips were incubated with 0.15% glutaraldehyde/phosphate-buffered saline (PBS) for 10 min followed by PBS washes. A total of 100 μL of 1:9 FITC–gelatin (Invitrogen): 0.2% porcine gelatin was added. Coverslips were then washed in PBS and incubated 15 min in 5 mg mL^−1^ NaBH_4_. Coverslips were rinsed in PBS and incubated at 37 °C in 10% FBS/DMEM for 2 h. MDA-MB-231 cells were seeded on coverslips with or without WJ460. Cells were incubated for 12 h and processed for immunofluorescence.

### Animal studies

Female nude mice BALB/c athymic nude mice (6–8 weeks) and BALB/c mice (6–8 weeks) were maintained in pathogen-free conditions with a 12 h light/dark cycle. Animal studies were performed according to the guidelines approved by the Institute of Biomedical Sciences, East China Normal University. For the spontaneous metastasis model, MDA-MB-231-Luciferase cells (1 × 10^6^) were injected in the #4 mammary fat pad of the mice. Tumor volume and spontaneous distant metastases were measured by a Xenogen IVIS 2000 Luminal Imager. For the experimental metastasis model, MDA-MB-231-Luciferase or MDA-MB-231-Luciferase-GFP cells (1 × 10^6^) were intravenously introduced into the mice. The bioluminescence was monitored weekly.

### RTK array

Human phosphorylated RTK array was performed according to the manufacturer’s instruction (R&D Systems™ Proteome Profiler Human Phospho-RTK Array Kit, Catalog # ARY001B). Briefly, 800 μg of cell lysate from MDA-MB-231 or BT549 cells was incubated on the membrane spotted with capture antibodies. After extensive washing, the anti-phosho-tyrosine-horseradish peroxidase antibody was added and images were acquired by the Chemi Reagent Mix.

### Streptavidin–biotin affinity pull-down assay

Cell lysates were harvested and incubated with free biotin or WJ460–biotin at 4 °C with gentle rotation. Recombinant streptavidin agarose beads were subsequently added to precipitate proteins interacting with biotin-labeled WJ460. The bead-bound proteins were separated by SDS-PAGE and visualized by Silver Staining. The indicated protein-containing band was cut out for mass spectrometry.

### Immunofluorescence assay

Fixed cells were permeabilized with 0.1% Triton X-100 in PBS and blocked with 1% bovine serum albumin followed by incubation with the indicated antibodies overnight. Cells were then stained with secondary antibodies. 4,6-Diamidino-2-phenylindole was used to visualize the nuclei. Fluorescence signals were acquired on a confocal microscope (Leica).

### Tissue array and immunohistochemical (IHC) staining

Breast cancer and normal tissue microarray sections were obtained from Alena Biotechnology Co, Ltd, China. This tissue array contains tissues from 4 cases of stage I breast carcinoma, 40 cases of stage II breast carcinoma, 5 cases of stage III breast carcinoma, 1 case of stage IV breast carcinoma, 40 cases of metastatic invasive breast ductal carcinoma, and 10 cases of adjacent chronic mastitis. This array was used for IHC staining.

### Immunoblotting analysis

Cell lysates were lysed in RIPA buffer and NP-40 lysis buffer (50 mM Tris pH 8.0, 150 mM NaCl, 1% NP-40, 0.5 mM EDTA, 10% Glycerol) containing protease inhibitors and phosphatase inhibitors and then analyzed by immunoblotting with the indicated antibodies. All the antibodies used in our studies are described in Supplementary Table 2. Uncropped western blots are shown in Supplementary Fig. 13.

### Construction of MYOF expression vectors

Construct containing human MYOF C2A-C (F1) encodes amino acids 1–535. Construct containing human MYOF FerA, FerB, and DsyF (F2) represents amino acids 535–1116. C2D containing construct (F3) encodes amino acids 1116–1272. C2E-F containing construct (F4) encodes amino acids 1272–1930. Each insert was ligated into the BamH1 and Xho1 sites of pGEX4T-1. The inserts were transfected into BL21 cells and GST-MYOF fusion protein expression were induced by isopropyl 1-thio-β-D-galactopyranoside. The primers are described in Supplementary Table 3.

### Surface plasmon resonance

SPR was determined using a Biacore X-100 plus instrument (GE). MYOF C2D peptides were immobilized on the sensor chip (CM5) using the amine-coupling method according to standard protocols. MYOF C2D peptide was diluted in sodium acetate buffer, pH 4.5. WJ460 was diluted to 0.1–5 μM in PBS. To estimate the affinity, the binding assay was examined at 25 °C at a flow rate of 30 μL min^−1^ using PBS buffer. The affinity constants of binding were obtained using 1:1 Langmuir binding model via BIAevaluation software.

### Knockdown of MYOF by siRNA and shRNA

Lipofectamine-mediated transient transfection of MYOF siRNA was performed. shRNA directed against human MYOF was inserted into the pLKO.1 vector. 293T cells were transiently transfected with the following plasmids: lentiviral packaging plasmid pMD2.G, psPAX2, and lentiviral expression plasmid. Forty eight hours post-transfection, the supernatant was harvested. The sequences of siRNA and shRNA against MYOF used in our studies are described in Supplementary Table 3.

### Statistical analysis

Grouped data are expressed as mean ± s.d. Significance between groups was analyzed by one-way analysis of variance or Student’s *t* test using GraphPad Prism 5.0 (GraphPad Software). All experiments were performed at least three times except for animal experiments. *p* Values <0.05 were considered statistically significant. Animal experiment sample size was chosen to ensure adequate statistical power (>80%) according to formal power calculation.

## Electronic supplementary material

Supplementary Information

## Data Availability

The authors declare that the data supporting this study are available within the paper and its Supplementary Information File. All other data are available from the authors upon reasonable request.
