# Supplementary material for: A small molecule targeting myoferlin exerts promising anti-tumor effects on breast cancer
Source: Nat Commun. 2018 Sep 13;9:3726. doi: 10.1038/s41467-018-06179-0 (PMC6137146; doi:10.1038/s41467-018-06179-0)
Supplement: Supplementary file 1 — Supplementary Information [file 41467_2018_6179_MOESM1_ESM.pdf]

A small molecule targeting myoferlin exerts promising anti-tumor effects on breast cancer

Tao Zhang et al.

a

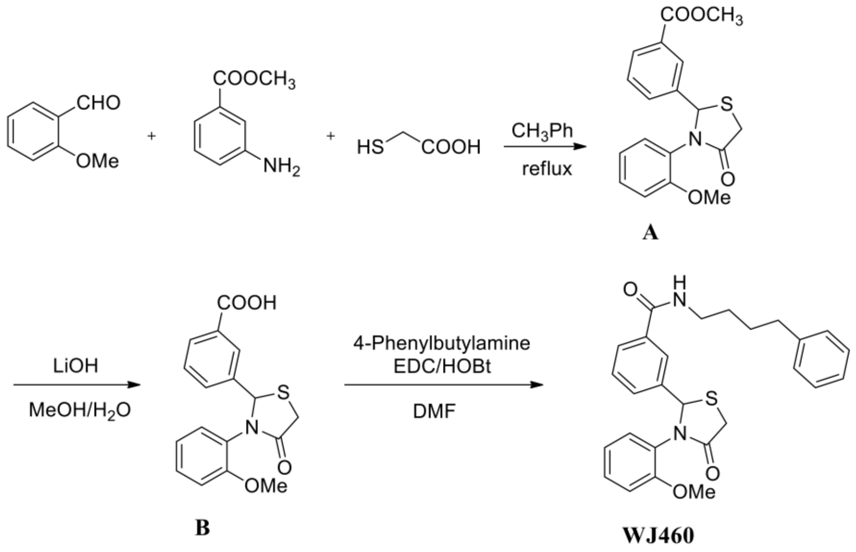

The synthesis scheme of WJ460

b

Acquisition Parameter

Source Type	ESI	Ion Polarity	Positive	Set Nebulizer	1.5 Bar
Focus	Active	Set Capillary	3700 V	Set Dry Heater	180 °C
Scan Begin	50 m/z	Set End Plate Offset	-500 V	Set Dry Gas	6.0 l/min
Scan End	1200 m/z	Set Collision Cell RF	500.0 Vpp	Set Divert Valve	Waste

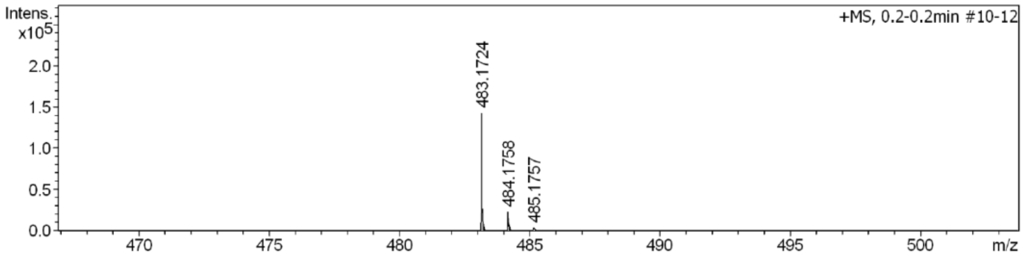

#	m/z	Res.	S/N	I	I %	FWHM
1	483.1724	33691	4267.7	142252	100.0	0.0143
2	484.1758	24937	680.1	22670	15.9	0.0194
3	485.1757	15699	93.7	3120	2.2	0.0309

Meas. m/z	#	Ion Formula	m/z	err [ppm]	mSigma		Score	rdb	e⁻ Conf	N-Rule
483.1724	1	C27H28N2NaO3S	483.1713	-2.3	85.5	9	12.08	14.5	even	ok

Supplementary Figure 1 (**a**) The synthesis scheme of WJ460. The intermediate methyl 3-(3-(2-methoxyphenyl)-4-oxo-2-thiazolidinyl)benzoate (A), constructed from 2-methoxybenzaldehyde, methyl 3-aminobenzoate and thioglycolic acid, was hydrolyzed with LiOH·H_2O to yield 3-(3-(2-methoxyphenyl)-4-oxo-2-thiazolidinyl) benzoic acid (B), and the key intermediate B was treated with EDC·HCl, HOBt and 4-phenylbutylamine in anhydrous DMF to get the targeted compound 3-(3-(2-methoxyphenyl)-4-oxo-2-thiazolidinyl)-*N*-(4-phenylbutyl) benzamide (WJ-460). HPLC purity: 99%. All reactions were monitored by TLC and UV light, column

chromatography was performed on silica gel, HPLC (Agilent Technologies 1200 Series) was utilized for purity, injection volume was 10 μL, flow rate of 1.5 mL/min, solvent A: H_2O; solvent B: MeOH; gradient of 40−90% B (0−10 min), 90% B (10−15 min), 90−40% B (15−20 min). (**b**) The high-resolution mass spectrum (HR MS) of WJ460. HRMS (ESI): calcd for $[C_{27}H_{28}N_2O_3S + Na]^+$ 483.1713, found 483.1724. High-resolution mass spectrum (HRMS) was gathered on a Bruker MicroTOF-Q II LCMS instrument operating in electrospray ionization (ESI).

a

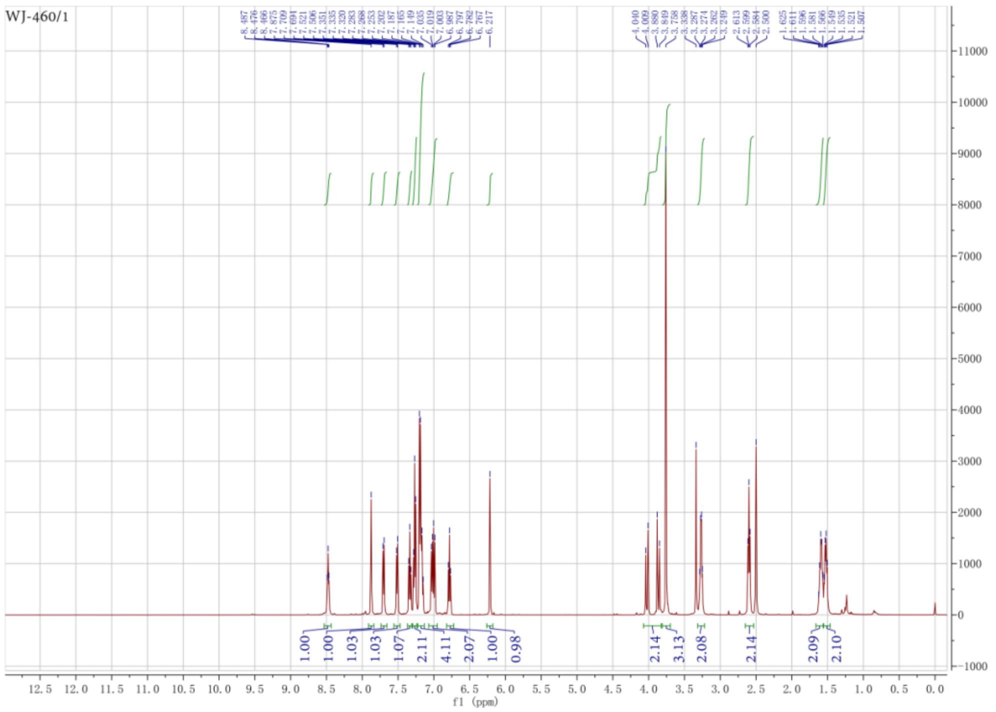

b

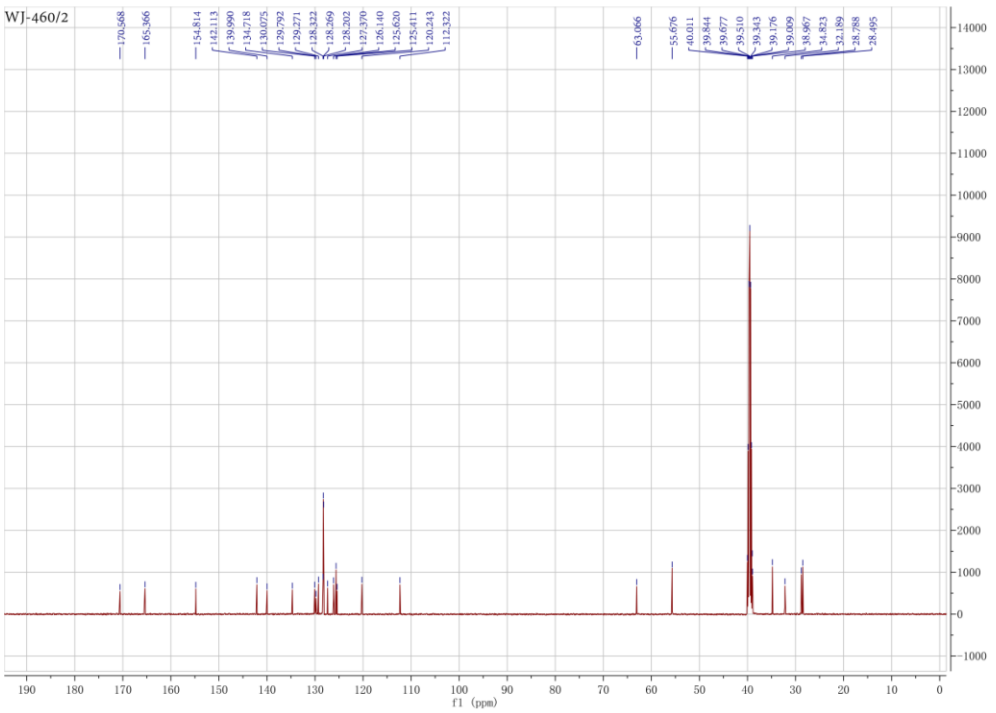

Supplementary Figure 2 **(a)** ^{1}H NMR spectrum of WJ460 (DMSO-d_6, 500 MHz): δ 8.64 (t, *J* = 5.5 Hz, 1H), 7.88 (s, 1H), 7.70 (d, *J* = 7.5 Hz, 1H), 7.51 (d, *J* = 7.0 Hz, 1H), 7.34 (dd, *J* = 7.5, 7.5 Hz, 1H), 7.27 (dd, *J* = 7.5, 7.5 Hz, 2H), 7.20-7.15 (m, 4H), 7.01 (dd, *J* = 8.0, 8.0 Hz, 2H), 6.78 (t, *J* = 5.0 Hz, 1H), 6.22 (s, 1H), 4.04-3.85 (m,

2H), 3.76 (s, 3H), 3.29-3.25 (m, 2H), 2.61-2.58 (m, 2H), 1.63-1.57 (m, 2H), 1.55-1.51 (m, 2H). (**b**) ^{13}C NMR spectrum of WJ460 (DMSO-d_6, 125 MHz): δ 170.57, 165.37, 154.81, 142.11, 139.99, 134.72, 130.08, 129.79, 129.27, 128.32, 128.27, 128.20, 127.37, 126.14, 125.62, 125.41, 120.24, 112.32, 63.07, 55.68, 38.97, 34.82, 32.19, 28.79, 28.49. NMR spectra were recorded on a Bruker 500 MHz instrument and obtained as DMSO-d_6 solutions (reported in ppm).

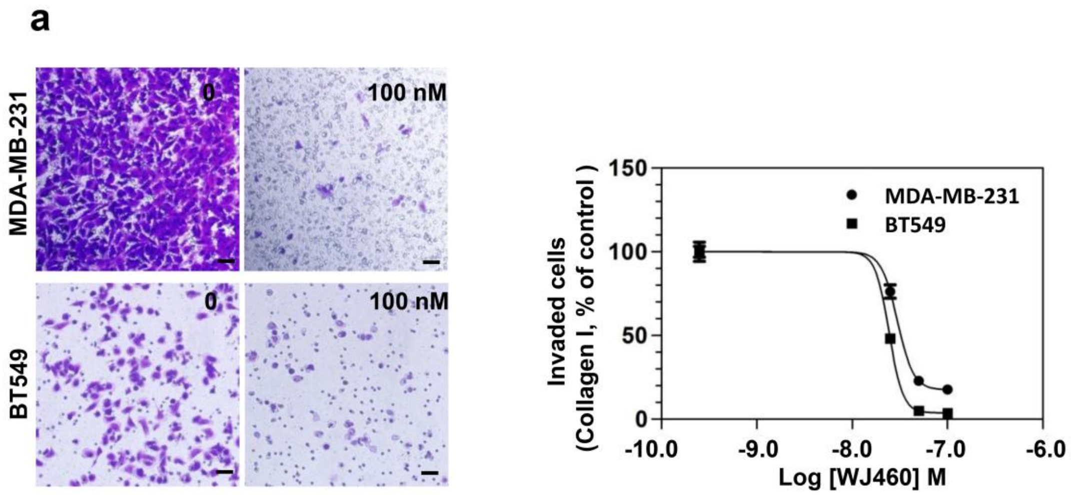

Supplementary Figure 3 (**a**) WJ460 impaired breast cancer cell invasion through collagen I. MDA-MB-231 and BT549 cells were seeded into each transwell insert precoated with 10% collagen I. After 12 h, invading cells were stained with 0.1% crystal violet. Images were acquired using an inverted microscope (Olympus). WJ460 inhibits MDA-MB-231 and BT549 cell invasion through collagen I with IC_{50} of 29.7 ±1.18 nM and 24.3±0.92 nM, respectively (n = 3). Scale bars, 50 μm.

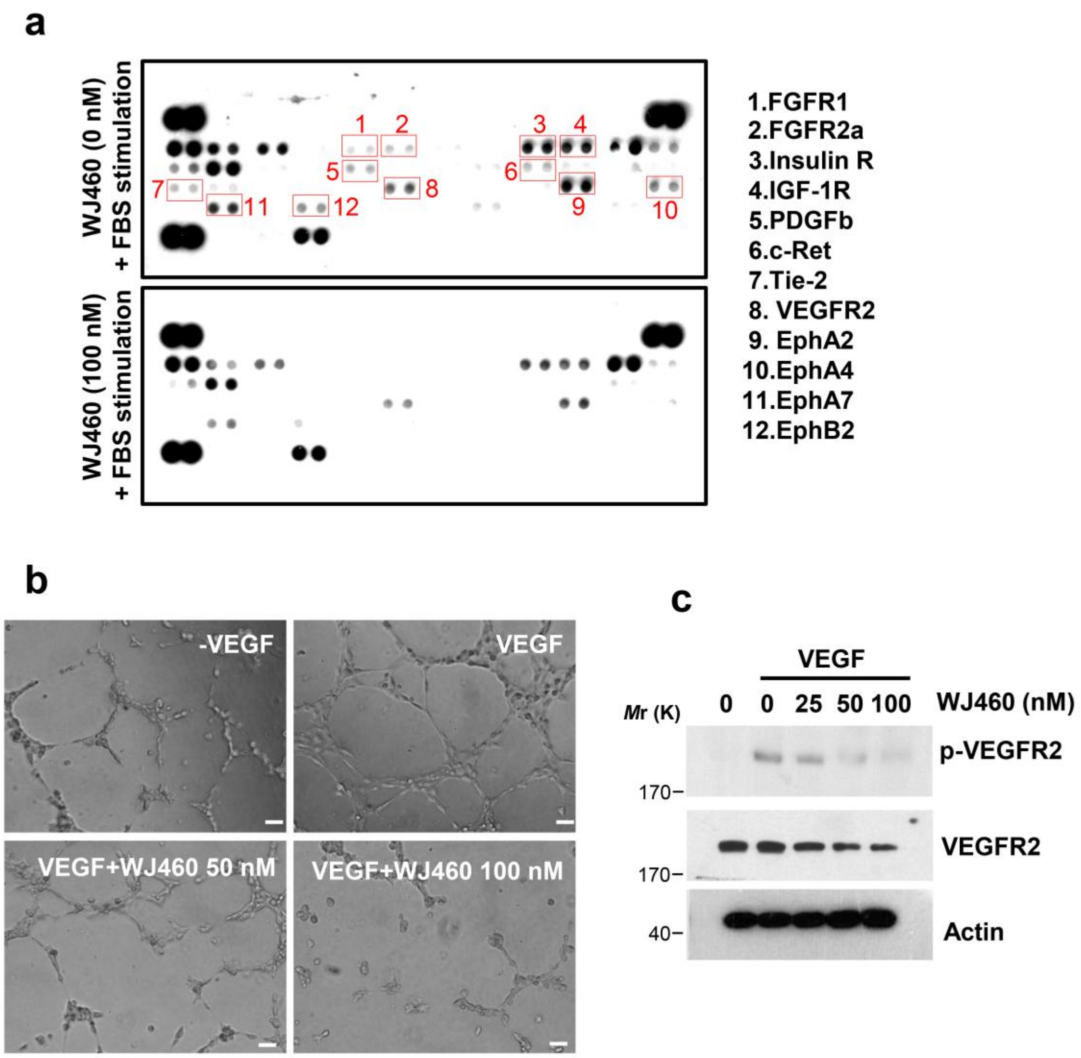

Supplementary Figure 4 WJ460 blocked receptor tyrosine kinases activities and VEGF-induced angiogenesis. (**a**) Screening of signaling pathways inhibited by WJ460 in MDA-MB-231 metastatic breast cancer cells by probing a human phospho-tyrosine kinase array with lysates from cells stimulated by FBS (10%) in the presence or absence of WJ460 (100 nM). Proteins showing decreased phosphorylation upon WJ460 treatment are highlighted. (**b**) HUVECs (5×10^4) were seeded in Matrigel-coated wells for 6 hours in the presence or absence of WJ460 (100 nM) and VEGF (100 ng/mL). Cells were fixed, and tubular-like structures were photographed. (**c**) HUVECs were pretreated with WJ460 and stimulated with VEGF (100 ng/mL) (*n* = 3). Scale bars, 20 μm. Proteins were extracted and subjected to immunoblotting with indicated antibodies (*n* = 3). In **b** and **c**, *n* indicates the number of independent experiments performed.

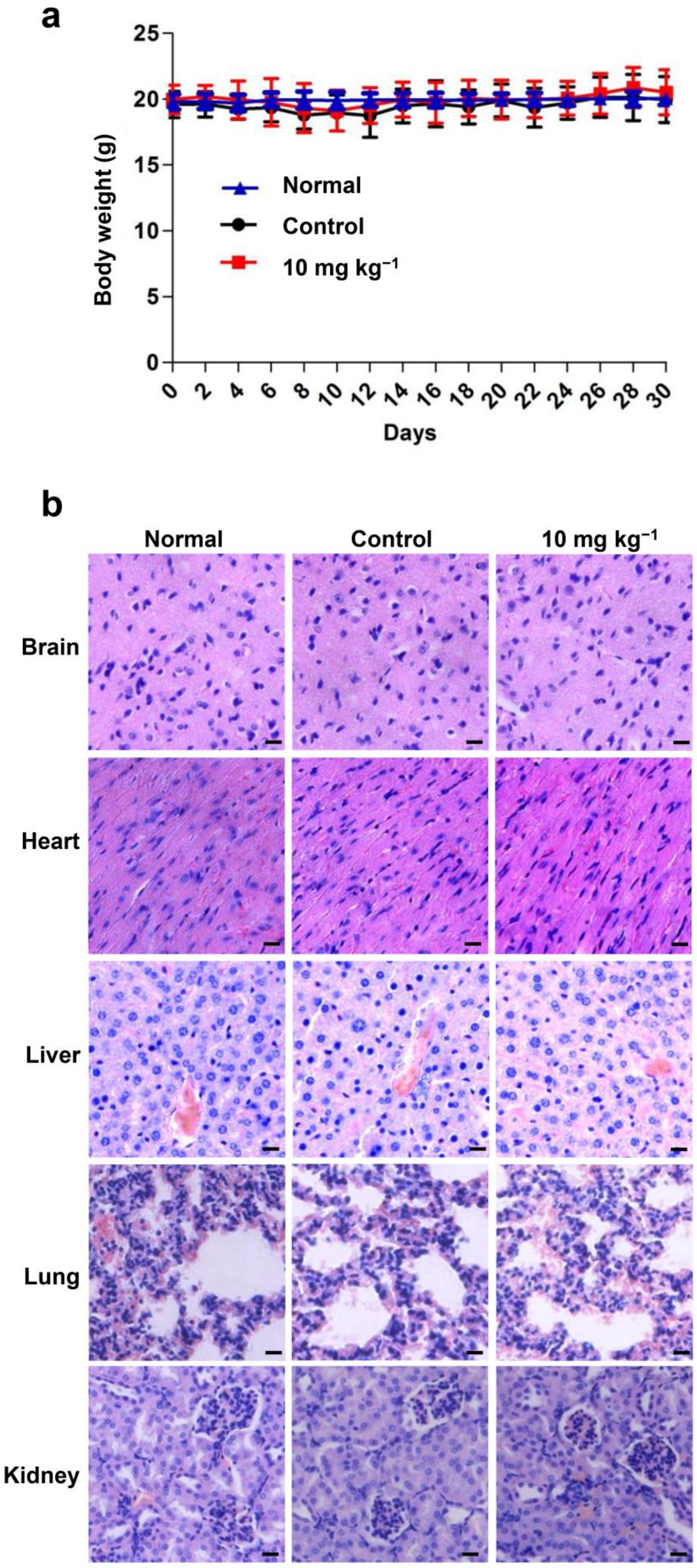
a
Body weight (g)
25
20
15
10
5
0
Normal
Control
10 mg kg⁻¹
0
2
4
6
8
10
12
14
16
18
20
22
24
26
28
30
Days
b
Normal
Control
10 mg kg⁻¹
Brain
Heart
Liver
Lung
Kidney

Supplementary Figure 5 WJ460 showed little toxicity in mice. (**a**) 8 week-old female BALB/c mice were left untreated (Normal) or treated with DMSO (Control) or WJ460 (i.p.) for 30 days. Mice body weight was measured every other day. (**b**) When the experiment terminated, major organs in each group were freshly dissected, fixed and paraffin-embedded. H&E staining of sections followed by histopathological analysis was performed. Scale bars, 20 μm.

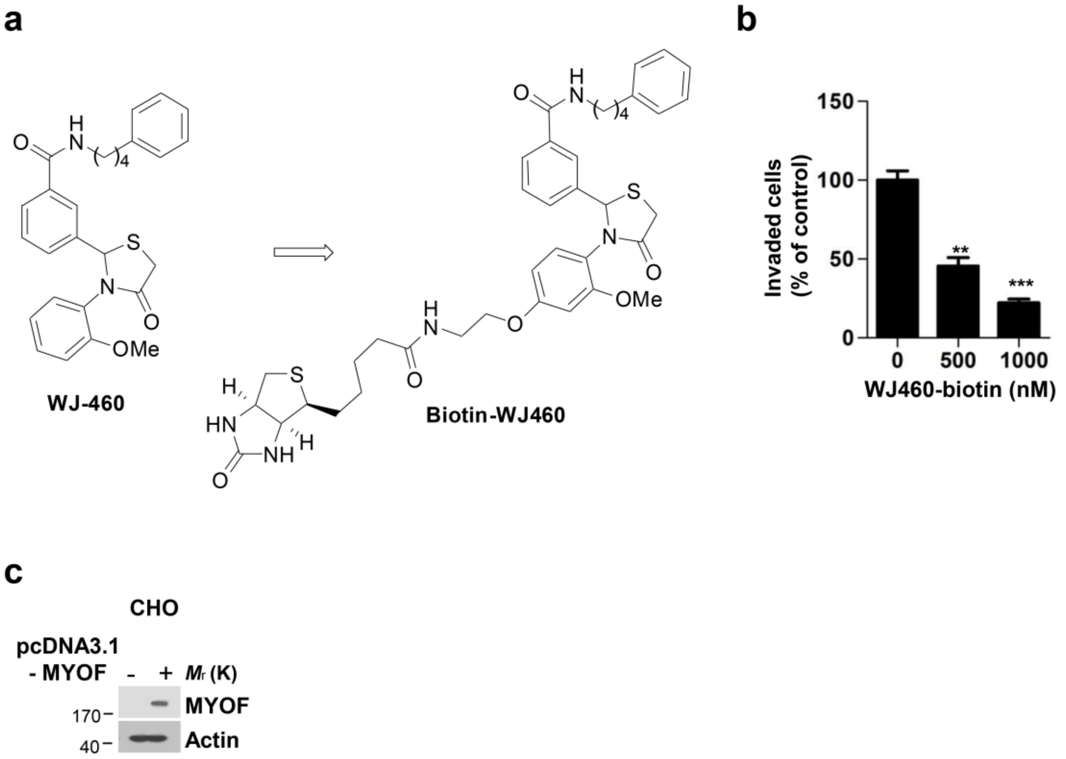

Supplementary Figure 6 Myoferlin is a target of WJ460. (**a**) Chemical structure of WJ460 and WJ460-biotin. (**b**) WJ460-biotin suppressed MDA-MB-231 cell invasion, Data shown are mean ± s.d. ** $p < 0.01$, *** $p < 0.001$. Student's t test, $n = 3$. (**c**) CHO cells were forced to express human myoferlin, cell lysates were extracted and detected by western blot ($n = 3$). In **b** and **c**, n indicates the number of independent experiments performed.

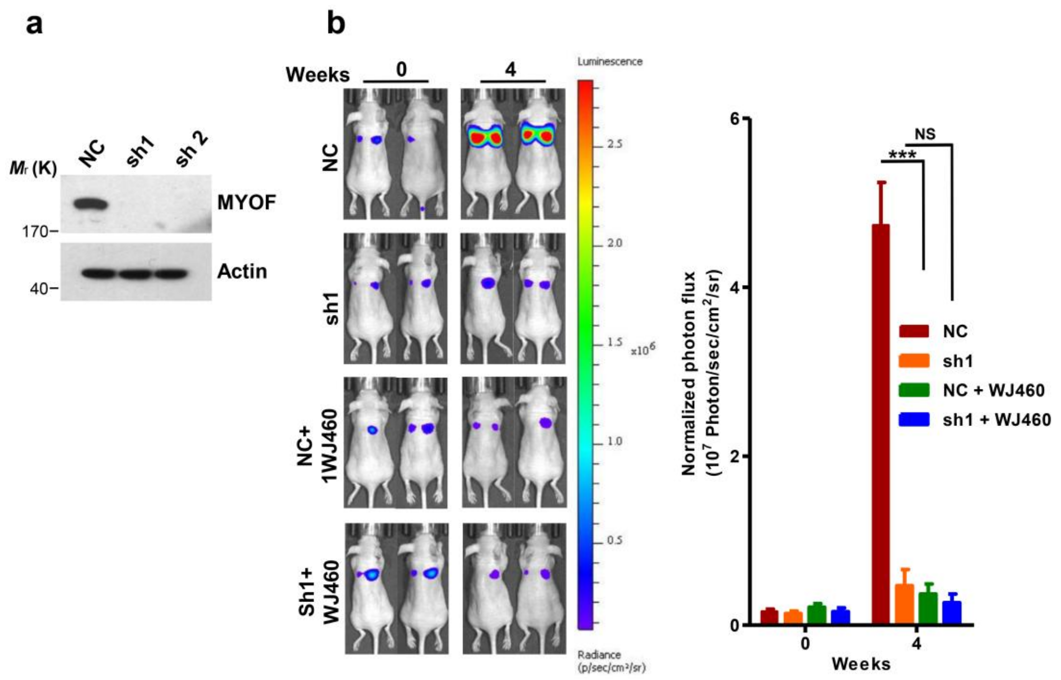

Supplementary Figure 7 Myoferlin knockdown in MDA-MB-231-Luciferase cells impaired lung metastases. (**a**) Myoferlin was knocked down by specific shRNAs in MDA-MB-231-Luciferase cells (n = 3). (**b**) Myoferlin knockdown in MDA-MB-231-Luciferase cells rendered cancer cells resistant to WJ460 treatment in animal model. Cells were transfected with scrambled shRNA or MYOF shRNA, and then were inoculated intravenously into female nude mice. DMSO or WJ460 was injected intraperitoneally.

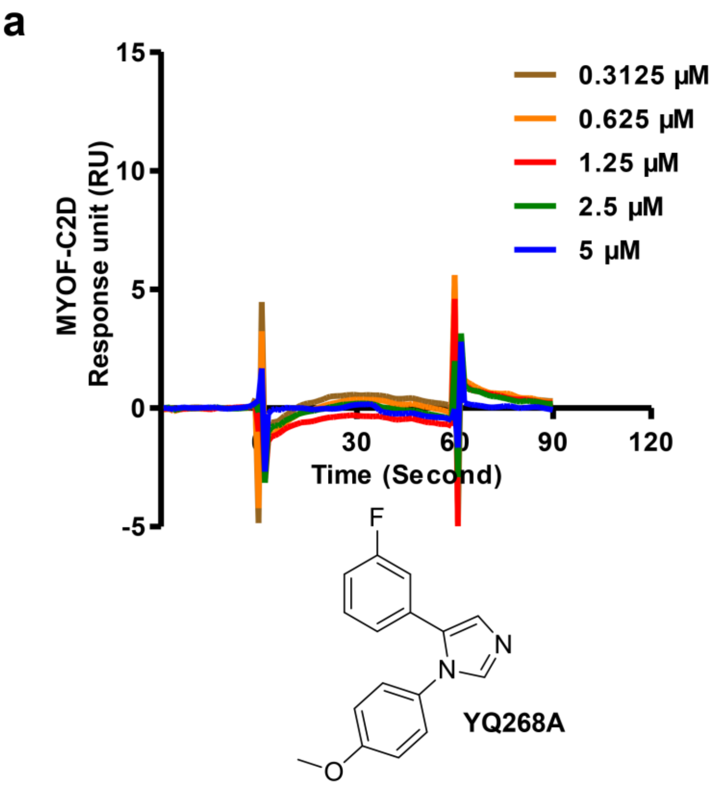

Supplementary Figure 8 (**a**) The binding between a negative molecule (YQ268A) and MYOF C2D domain was examined by surface plasmon resonance (SPR) assay.

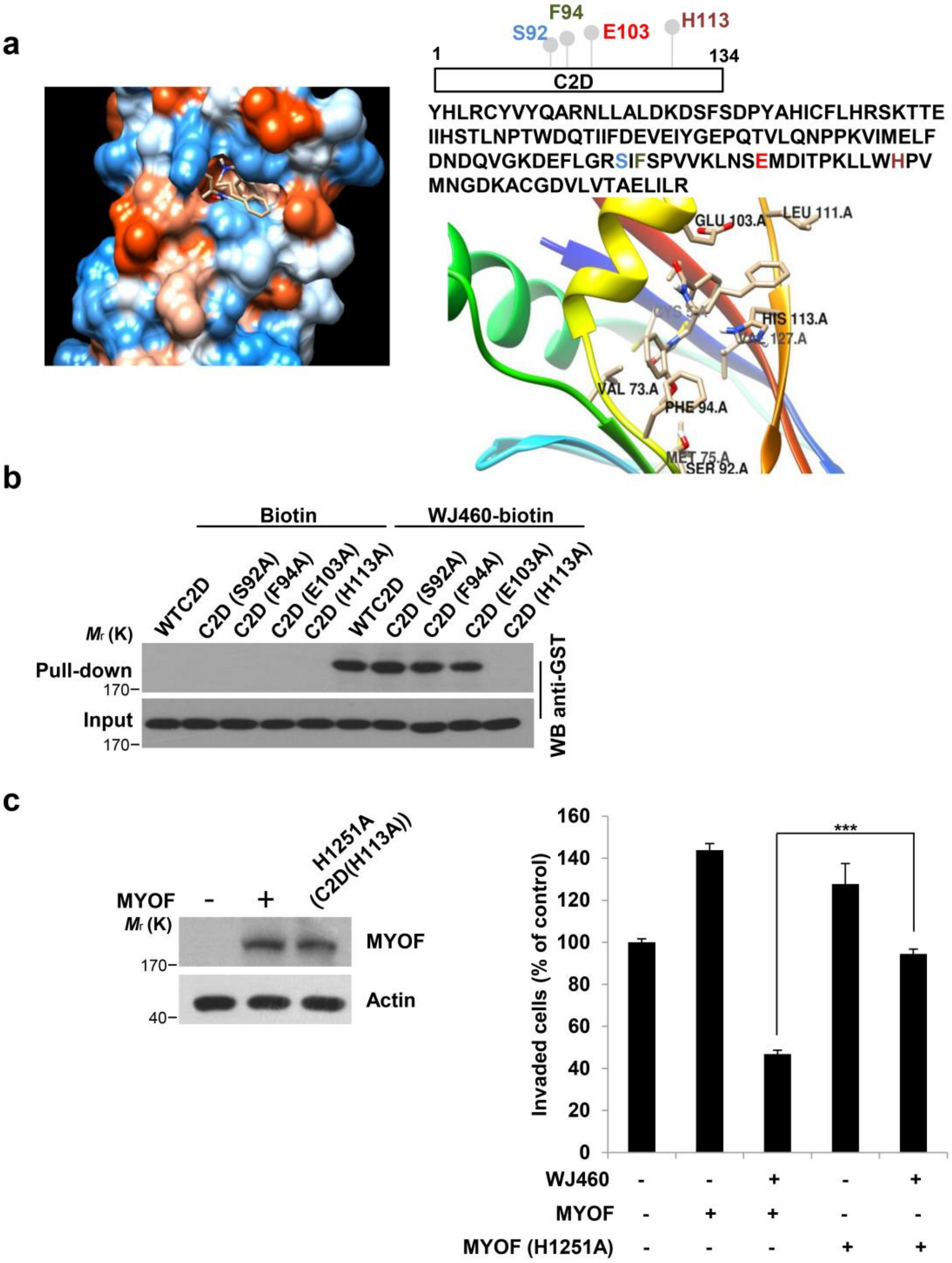

Supplementary Figure 9 C2D domain of MYOF is responsible for its interaction with WJ460. (**a**) Binding model of WJ460 in MYOF-C2D (left). A homology modeling assay was performed and the potential amino acid residues responsible for the binding between WJ460 and C2D domain were highlighted. (**b**) Recombinant wild-type (WT) MYOF-C2D and its mutants were incubated with biotin or WJ460-biotin, followed by pull down assay. (**c**) Boyden-chamber cell invasion assay of

MDA-MB-231cells transfected with MYOF or mutants of MYOF vector in the presence or absence of WJ460 (100 nM).

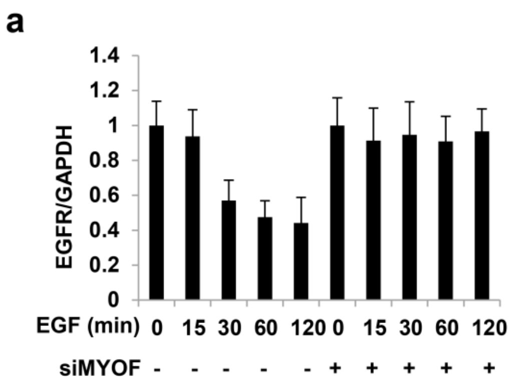

Supplementary Figure 10 The EGFR bands in Figure5 d and e were quantified and normalized according GAPDH, n = 3.

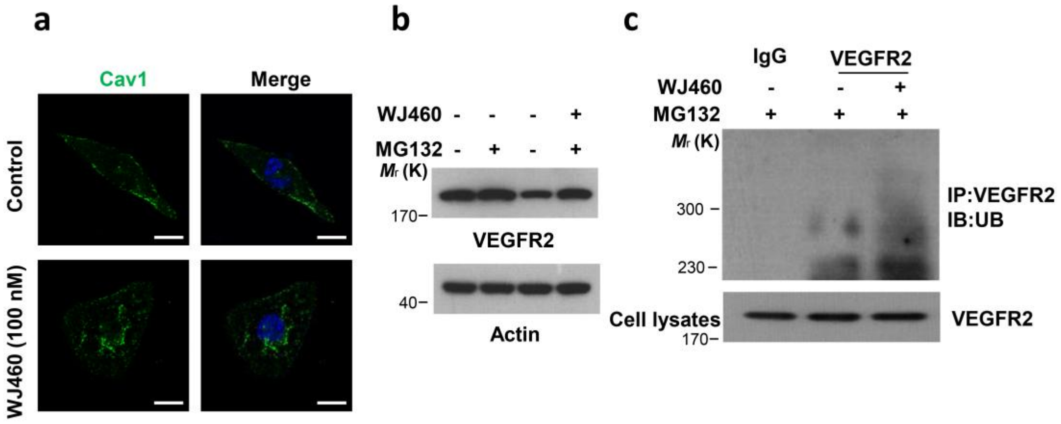

Supplementary Figure 11 WJ460-MYOF interaction hampers the proper function of MYOF. (**a**) Immunofluorescence analysis of Cav1 in WJ460 treated MDA-MB-231 cells 120 min post-EGF stimulation. scale bars, 10 μm. (**b**) HUVECs cells were treated with WJ460 (100 nM) with or without MG132 (10 μM) and VEGFR2 protein level was measured by western blotting analysis. (**c**) Immunoprecipitation (IP) was performed using anti-VEGFR2 and immunoblotting performed with an anti-ubiquitin antibody.

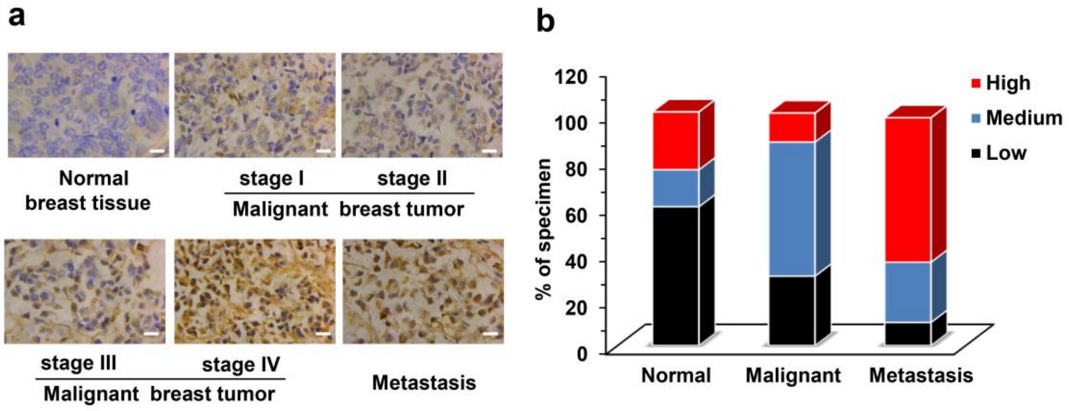

Supplementary Figure 12 A role of MYOF in the pathogenesis of breast cancer. (**a**) a Representative immunohistochemical (IHC) staining of MYOF in 90 human breast cancer biopsy samples and 10 adjacent non-transformed specimens. Scale bar, 50 μm. (**b**) The proportion of MYOF expression level was calculated in 3 different breast cancer stage samples (normal, malignant and metastasis).

Figure 4

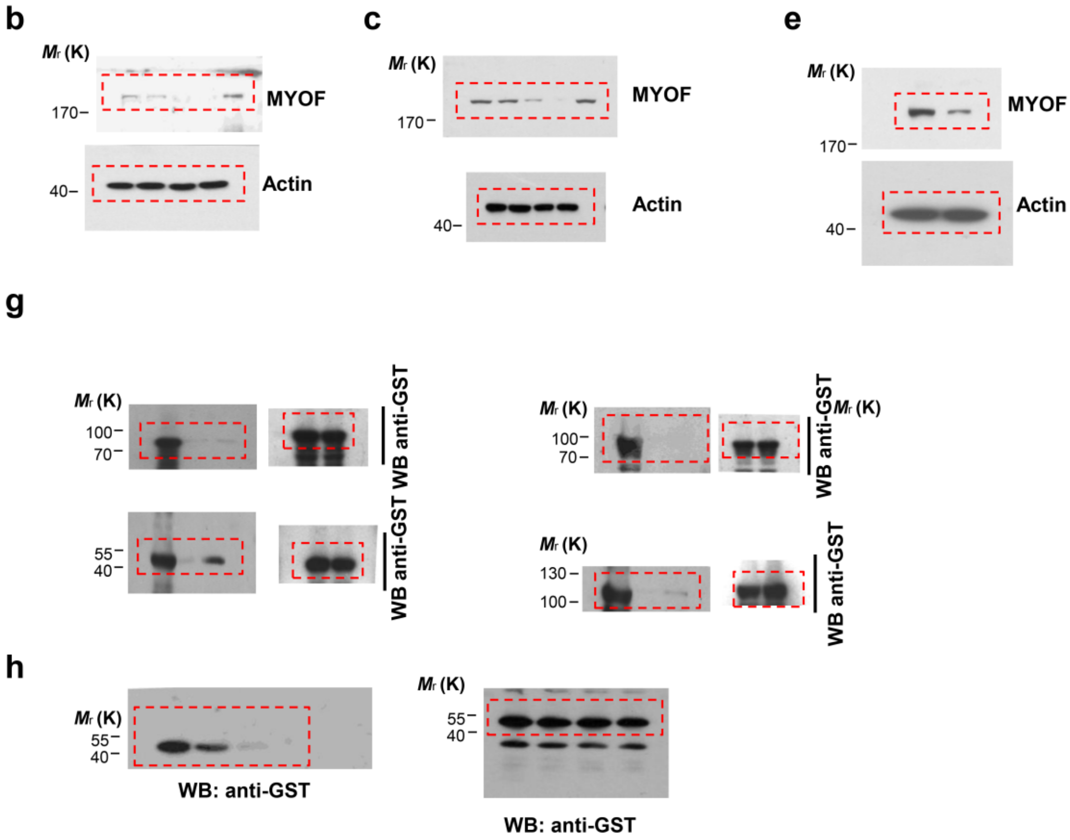
b
Mr (K)
170
MYOF
40
Actin
c
Mr (K)
170
MYOF
40
Actin
e
Mr (K)
170
MYOF
40
Actin
g
Mr (K)
100
70
WB anti-GST
55
40
WB anti-GST
Mr (K)
100
70
WB anti-GST
Mr (K)
Mr (K)
130
100
WB anti-GST
h
Mr (K)
55
40
WB: anti-GST
Mr (K)
55
40
WB: anti-GST

Figure 5

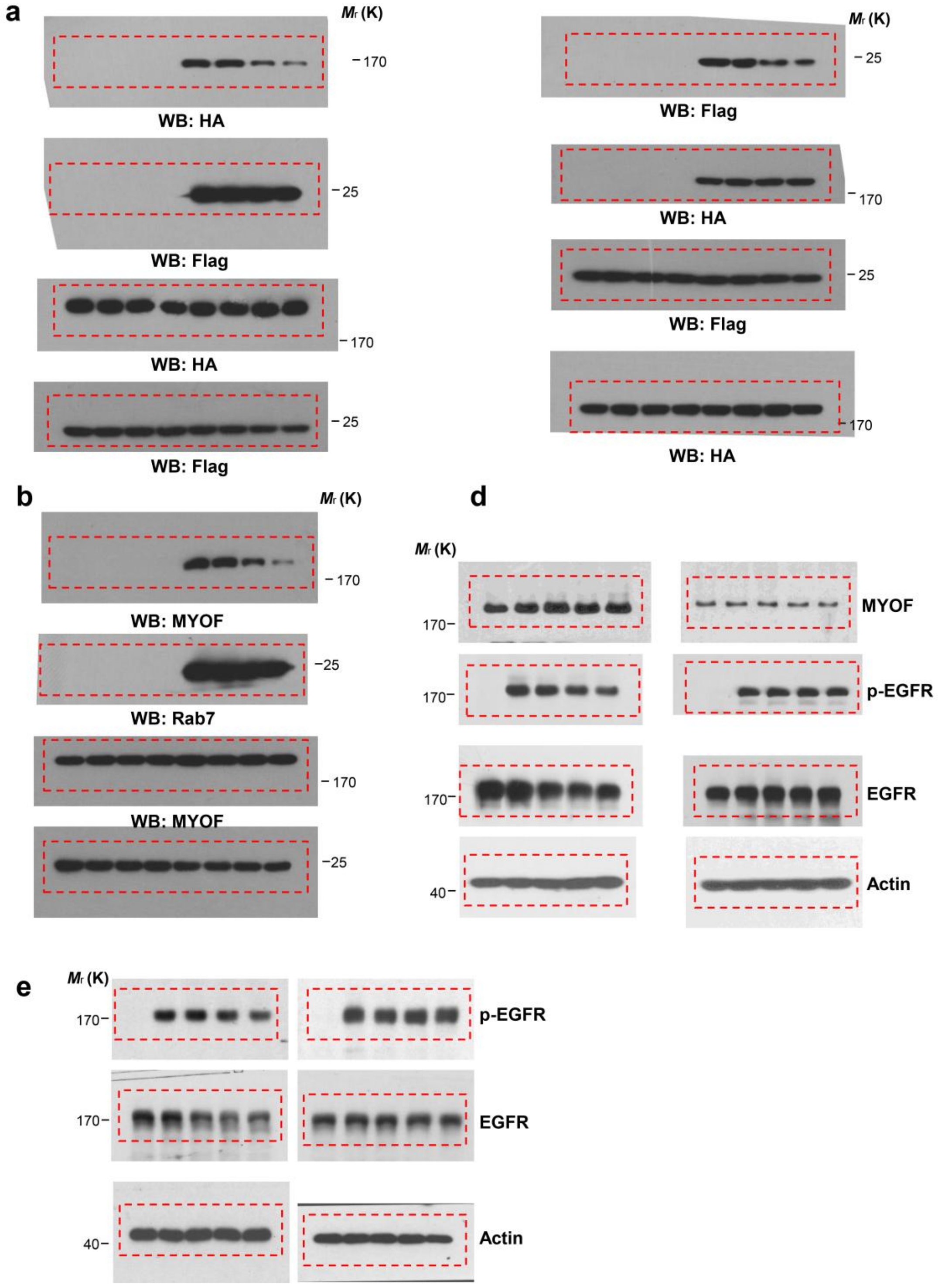
a
Mr (K)
170
WB: HA
25
WB: Flag
170
WB: HA
25
WB: Flag
Mr (K)
25
WB: Flag
170
WB: HA
25
WB: Flag
170
WB: HA
b
Mr (K)
170
WB: MYOF
25
WB: Rab7
170
WB: MYOF
25
d
Mr (K)
170
MYOF
170
p-EGFR
170
EGFR
40
Actin
e
Mr (K)
170
p-EGFR
170
EGFR
40
Actin

Figure 6

a

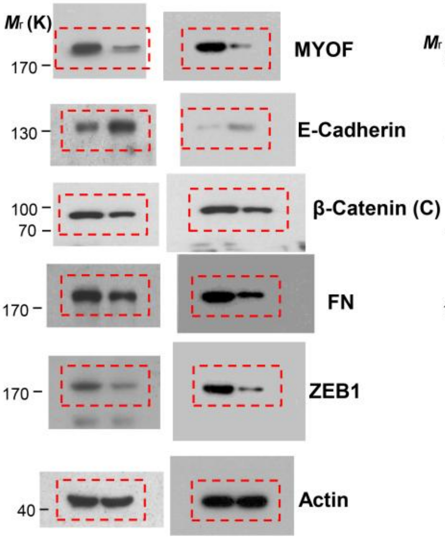
Mr (K)
170
130
100
70
170
170
40
MYOF
E-Cadherin
β-Catenin (C)
FN
ZEB1
Actin

b

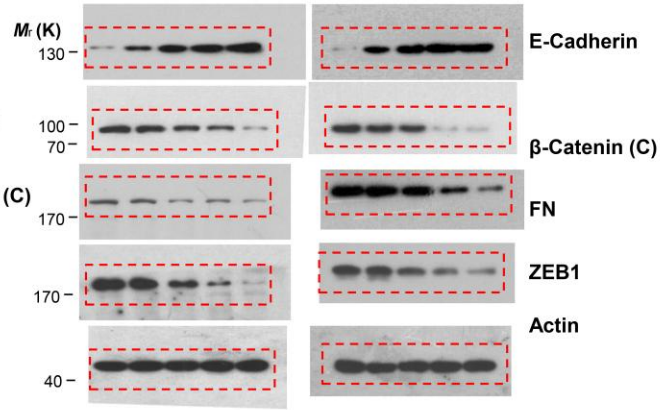
Mr (K)
130
100
70
170
170
40
E-Cadherin
β-Catenin (C)
FN
ZEB1
Actin

f

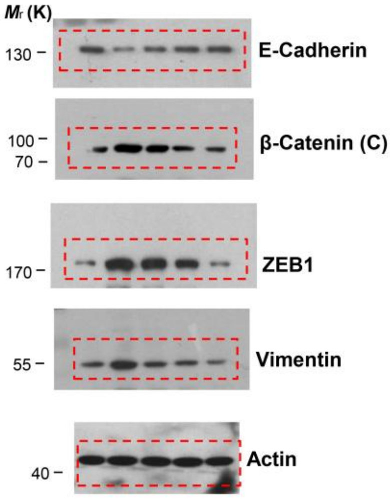
Mr (K)
130
100
70
170
55
40
E-Cadherin
β-Catenin (C)
ZEB1
Vimentin
Actin

Supplementary Figure 4

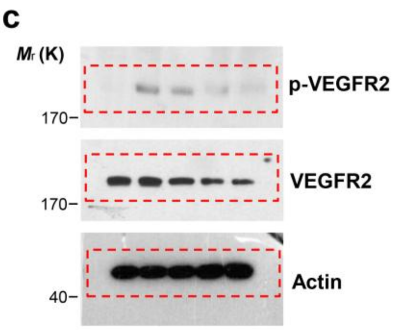

Supplementary Figure 6

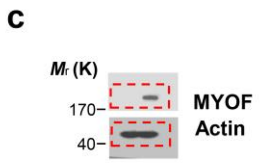

Supplementary Figure 7

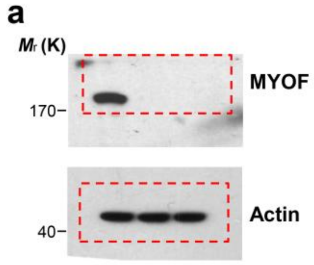

Supplementary Figure 9

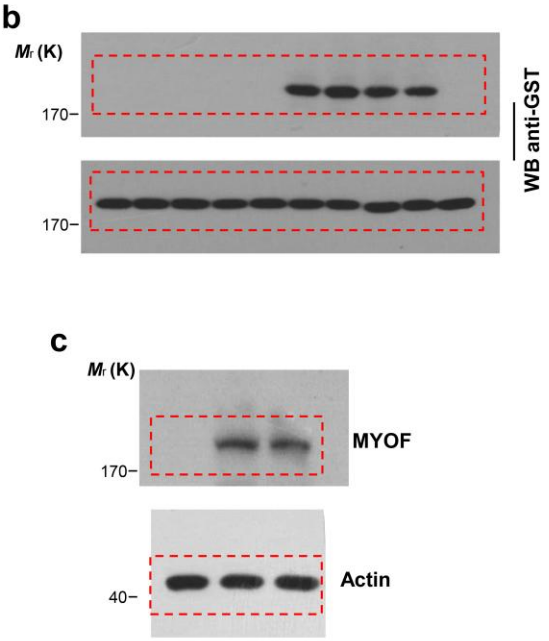

Supplementary Figure 11

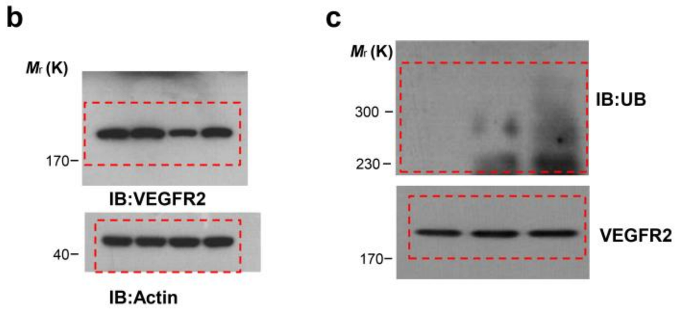

Supplementary Figure 13 Unprocessed original scans of blots.

Peptide sequence	MH+	DeltCN	xCorr
**MYOF isoform of Myoferlin [Homo sapiens]**			
R.GIPLDFSSSLGIIVK.D	1545.889	0.4138	2.2926
K.GPVGTVSEAQLAR.R	1284.6937	0.447	3.389
K.VSMFVLGTGDEPPPERR.D	1887.9474	0.2435	2.5386
R.DRDNDSDDVESNLLLPAGIALR.W	2398.19	0.2673	3.7722
R.AEDIPQMDDAFSQTVK.E	1794.8226	0.3574	2.2508
K.NLVDPFVEVSFAGK.K	1522.979	0.0491	3.0832
K.NDVVGTTYLHLSK.I	1446.764	0.1791	2.6768
R.ILVELATFLEK.T	1275.7585	0.1868	3.0928
K.LEPISNDDLLVVEK.Y	1583.8551	0.1886	2.3723
R.SLSQIHEAAVR.M	1210.6543	0.192	2.0355
R.IPAHQVLYSTSGENASGK.Y	1858.9323	0.2737	3.0761
R.SLLTEADAGHTEFTDEVYQNESR.Y	2613.179	0.4753	3.8001
R.YPGGDWKPAEDTYTDANGDK.A	2200.953	0.4963	4.3918
K.LEGALGADTTEDGDEK.S	1620.7266	0.2505	1.9474
K.HSATTVFGANTPIVSCNFDR.V	2194.0337	0.4253	3.2213
R.KPVVGQCTIER.L	1286.6886	0.1265	2.2279
R.DIVIEMEDTKPLLASK.L	1801.9656	0.2751	2.659
R.DIVIEMEDTKPLLASK.L	1801.966	0.1274	2.7698
R.GKSDENEDPSVVGEFK.G	1736.8033	0.3623	2.1178
R.GKSDENEDPSVVGEFK.G	1736.8035	0.2078	2.3651
R.IYPLPDDPSVPAPPR.Q	1633.8616	0.3849	2.826
R.ELPDSVPQECTVR.I	1529.7279	0.3224	2.6846
R.GLELQPQDNNGLCDPYIK.I	2075.9973	0.4505	2.8088
K.ISVYDYDTFTR.D	1379.6488	0.4023	3.3728
K.VGETIIDLENR.F	1258.666	0.3742	3.8018
R.FGSHCGIPEEYCVSGVNTWR.D	2356.0303	0.2608	2.3972
R.DQLRPTQLLQNVAR.F	1651.9253	0.3196	3.9068
R.FKGFPQPILSEDGSR.I	1678.8644	0.3798	2.7113

Supplementary Table 1 (Relevant to Figure 3a) Myoferlin is identified by LC-MS/MS.

Antibody	Vendor	Catalogue number	Application	Dilution
Ki67	Abcam	ab16667	IHC-P	1:100
CD31	Abcam	ab28364	IHC-P	1:50
MYOF	Abcam	ab76746	WB	1:1000
			IHC-P	1:100
Actin	Sigma	A5441	WB	1:10000
GST	CST	2624	IP	1:200
			WB	1:1000
HA	CST	3724	IP	1:50
			WB	1:5000
			IF	1:1600
Flag	CST	8146	IP	1:50
			WB	1:1000
			IF	1:1600
Rab7	CST	9367	IP	1:100
			WB	1:1000
EGFR	CST	4267	WB	1:1000
p-EGFR	CST	3777	WB	1:1000
E-cadherin	CST	3195	WB	1:1000
			IF	1:200
β-Catenin	CST	9562	WB	1:1000
Fibronectin	Abcam	ab137720	WB	1:1000
			IF	1:200
ZEB1	CST	3396	WB	1:1000
Vimentin	CST	5741	WB	1:1000
			IF	1:100
VEGFR2	CST	9698	WB	1:1000
			IP	1:100
p-VEGFR2	CST	2478	WB	1:1000
Ubiquitin	Abcam	ab134953	WB	1:1000
Caveolin-1	CST	3267	WB	1:1000
			IF	1:400

Supplementary Table 2 Information for antibodies.

PCR primers		
Gene		Sequence
CDH1	Forward	5’ CGAGAGCTACACGTTCACGG 3’
	Reverse	5’ GGGTGTCGAGGGAAAAATAGG 3’
FN	Forward	5’ GAGAATAAGCTGTACCATCGCAA 3’
	Reverse	5’ CGACCACATAGGAAGTCCCAG 3’
ACTB	Forward	5’ CATGTACGTTGCTATCCAGGC 3’
	Reverse	5’ CTCCTTAATGTCACGCACGAT 3’
MYOF siRNAs		
MYOF siRNA1		5’ CCCUGUCUGGAAUGAGA 3’
MYOF siRNA2		5’ CGGCGGAUGCUGUCAAAUA 3’
MYOF shRNAs		
MYOF shRNA1		5’ AGCTGATATTGATGGACAGTA 3’
MYOF shRNA2		5’ CGGGATGTTCACAACCGAATT 3’
MYOF expression constructs		
F1		5’ CGGGATTCCGTGCGAGTGATTGTGGAATCT 3’
		5’ CCGCTCGAGGCCTCTGTAGGCAACTCCTTC 3’
F2		5’ CGGGATTCCGAGGATCTTGGTTGAATTAG 3’
		5’ CCGCTCGAGGTGCTTCTGTTTCTCCAGGCT 3’
F3		5’ CGGGATTCCGAGTGCCACCACTGTGTTCGGAG 3’
		5’ CCGCTCGAGCCTCAGAATCAGCTCTGCAGTT 3’
F4		5’ CGGGATTCCGGGCAAGGATGGCTCCAAC 3’
		5’ CCGCTCGAGCATGGCTTTGAGGTCCGGAAT 3’

Supplementary Table 3 Information for PCR primers and the sequences of siRNAs and shRNAs.
